# Genomic epidemiology of *Cryptococcus* yeasts identifies adaptation to environmental niches underpinning infection across an African HIV/AIDS cohort

**DOI:** 10.1111/mec.13891

**Published:** 2016-11-08

**Authors:** Mathieu Vanhove, Mathew A. Beale, Johanna Rhodes, Duncan Chanda, Shabir Lakhi, Geoffrey Kwenda, Sile Molloy, Natasha Karunaharan, Neil Stone, Thomas S. Harrison, Tihana Bicanic, Matthew C. Fisher

**Affiliations:** ^1^ Department of Infectious Disease EpidemiologySt Mary's HospitalImperial College LondonLondonW2 1PGUK; ^2^ Institute of Infection and ImmunitySt. George's University of LondonBlackshaw RoadLondon SW17 0QTUK; ^3^ Division of Infection & ImmunityUniversity College LondonGower StLondon WC1E 6BTUK; ^4^ School of MedicineUniversity of ZambiaNationalist RoadPO Box 50110LusakaZambia; ^5^ Department of Biomedical SciencesUniversity of ZambiaPO Box 32379LusakaZambia

**Keywords:** *Cryptococcus neoformans*, ecological genetics, fungi, microbial ecology, niche modelling

## Abstract

Emerging infections caused by fungi have become a widely recognized global phenomenon and are causing an increasing burden of disease. Genomic techniques are providing new insights into the structure of fungal populations, revealing hitherto undescribed fine‐scale adaptations to environments and hosts that govern their emergence as infections. Cryptococcal meningitis is a neglected tropical disease that is responsible for a large proportion of AIDS‐related deaths across Africa; however, the ecological determinants that underlie a patient's risk of infection remain largely unexplored. Here, we use genome sequencing and ecological genomics to decipher the evolutionary ecology of the aetiological agents of cryptococcal meningitis, *Cryptococcus neoformans* and *Cryptococcus gattii*, across the central African country of Zambia. We show that the occurrence of these two pathogens is differentially associated with biotic (macroecological) and abiotic (physical) factors across two key African ecoregions, Central Miombo woodlands and Zambezi Mopane woodlands. We show that speciation of *Cryptococcus* has resulted in adaptation to occupy different ecological niches, with *C. neoformans* found to occupy Zambezi Mopane woodlands and *C. gattii* primarily recovered from Central Miombo woodlands. Genome sequencing shows that *C. neoformans* causes 95% of human infections in this region, of which over three‐quarters belonged to the globalized lineage VNI. We show that VNI infections are largely associated with urbanized populations in Zambia. Conversely, the majority of *C. neoformans* isolates recovered in the environment belong to the genetically diverse African‐endemic lineage VNB, and we show hitherto unmapped levels of genomic diversity within this lineage. Our results reveal the complex evolutionary ecology that underpins the reservoirs of infection for this, and likely other, deadly pathogenic fungi.

## Introduction

Pathogenic fungi are widely responsible for emerging infections in humans as well as plants and animals by expanding their host range, increasing their virulence or through invading novel environments (Giraud *et al*. 2010; Fisher *et al*. 2012). Understanding the adaptation of fungal pathogens to ecological niches and the hosts therein is central to understanding how fungi emerge as pathogens, thereby explaining destructive processes such as the global panzootics and pandemics that they cause (Fisher *et al*. 2012).

Two closely related species of pathogenic basidiomycete yeasts, *Cryptococcus neoformans* and *Cryptococcus gattii*, are the aetiologic agent of the cryptococcal disease in humans, and have emerged as significant pathogens both in time and space (Casadevall *et al*. 2003; Nielsen *et al*. 2007). Despite the increased availability of antiretroviral therapy, cryptococcal meningitis remains a neglected disease that is responsible for a large proportion of AIDS‐related deaths (Van Wyk *et al*. 2014), reaching 70% in healthcare‐deprived areas of sub‐Saharan Africa (Desalermos *et al*. 2012). The majority of clinical infections are caused by *C. neoformans* which accounts for 99% of the cases found worldwide (Chayakulkeeree & Perfect 2008; Hagen *et al*. 2015). Although this is largely related to these fungi infecting an increasing pool of highly susceptible HIV/AIDS patients, spatial emergence is ongoing and *C. gattii* has been the focus of interest since the occurrence of the well‐documented outbreak and spread across the Pacific Northwest of Canada and the USA (Hoang *et al*. 2004; Kidd *et al*. 2004). In common with many other opportunistic pathogenic fungi, *Cryptococcus* is a saprophyte and is acquired from the environment; infected patients are not known to onwardly transmit the organism. For this reason, in order to understand the epidemiology of human cryptococcosis it is necessary to describe the ecology that supports the environmental reservoirs of this infection (Hiremath *et al*. 2008). Desnos‐Ollivier *et al*. (2010) described the ecological niches of *C. neoformans* as ‘extraordinarily complex’, due to their probable inclusion of other fungi, bacteria, protists or viruses. Increasingly, the role of ecological interactions within microbial communities and how these relationships can alter species distribution on large geographical scales are being addressed (Johnson & Stinchcombe 2007), and it is time that integrative approaches such as these are applied to the study of pathogenic fungi as well as the diseases that they cause.

Hardin (1960) formalized the principle of competitive exclusion; this states that two species which are competing for the same resources, if ecological factors remain constant, cannot coexist and maintain populations of similar size. The realized niche represents the actual niche where a given species is present, constrained by biotic and abiotic pressures. The fundamental niche is the complete range that a species can potentially occupy (Hutchinson 1959; Connell 1961). Current hypotheses accounting for the worldwide dissemination of *C. neoformans* state that the species gained the ability to metabolize pigeon guano, which became its realized niche. These adaptations may then have led to this pathogens worldwide spread via the use of birds and their migratory routes as vectors; in this way, the pathogen thus ‘explored’ and exploited its fundamental niche (Casadevall & Perfect 1998; Granados & Castañeda 2005). In contrast, *C. gattii* is thought to be more restricted to its realized niche, which is believed to be sedentary trees (Nielsen *et al*. 2007).

Genetic analysis to date has shown that infection by *Cryptococcus* in Africa is largely caused by *C. neoformans* and infections by *C. gattii* are rarely recovered (Wiesner *et al*. 2012; Chen *et al*. 2015) *C. neoformans* can be broadly divided into three major lineages with the first two, VNI and VNII, being globally distributed whilst a third, VNB, appears to be restricted to southern Africa. Across this region, studies have shown that VNB is largely associated with the Mopane woodland ecoregion while VNI and VNII have a tendency to be associated with urban areas, bird guano and non‐Mopane tree‐types (Chen *et al*. 2015). The genetic lineage of *C. neoformans* appears to play an important role in infection, with infections by VNB appearing more virulent (Beale *et al*. 2015). Together, these studies suggest that the different species and lineages of *Cryptococcus* in Africa inhabit markedly different ecological niches and that this heterogeneity leads to variation in not only risk of infection for HIV/AIDS patients, but also their risk of developing severe disease (Beale *et al*. 2015).

In Zambia, cryptococcal meningitis remains a serious issue as the country has one of the highest burdens of HIV in the world, with a prevalence estimated at over 13% (UNAIDS 2015). The landscape of the country has changed rapidly over the last decades owing to intense deforestation, changes in human demography and the development of industrial agriculture (Chidumayo 2013). Thus, Zambia provides a broad set of spatial scales and dynamic ecosystems within which to study the physical (abiotic) and macroecological (biotic) factors affecting the *Cryptococcus* species complex in its natural environment. Being an opportunistic environmental pathogen, *Cryptococcus* is not only under spatial and climatic pressures but also under the influence other fungal species or predators. Our study aims to recover and map the aetiological agent of human cryptococcosis from the environment, in a setting where the burden of cryptococcal meningitis is the highest worldwide (UNAIDS 2015). In this study, we isolated *Cryptococcus* from across the two main ecoregions that dominate Zambia, the soils and trees of the dry tropical Miombo forests and their counterparts across the Zambezi Mopane Woodlands. We model the ecological niches within which the two species occurred and, in concert, investigate the soil fungal community associated with either *C. gattii* or *C. neoformans* using an *ITS2* metabarcoding approach to understand whether a particular microbial composition is associated with either pathogen. Finally, whole‐genome sequencing (WGS) was used to subsequently explore associations between environmental *Cryptococcus neoformans* genomes with those recovered from patients participating in an ongoing clinical trial in the capital city Lusaka in order to understand whether clinical isolates are associated with a particular genotype. Together, these data form a mosaic that reveals how physical and ecological factors structure the environmental diversity of *Cryptococcus* across the Zambian landscape, leading to infections in the people that find their home there.

## Materials and methods

### Environmental sampling

To determine the prevalence of *Cryptococcus neoformans* in Zambia, samples were collected during both the wet and dry seasons (*n *=* *1356). Environmental sampling took place in January 2013 at the beginning of the rainy season, and a total of 583 samples were collected from soils, tree bark, pigeon guano and insects, substrates from which *Cryptococcus* species have been commonly isolated (Granados & Castañeda 2005; Litvintseva *et al*. 2005; Randhawa *et al*. 2005). The dry season was sampled in September 2013, and 773 samples were collected. Tree species were recorded, and tree tags were used with GPS coordinates in order to be able to return to each site. Two ecoregions were investigated throughout Zambia namely Central Miombo woodlands and the Zambezi and Mopane woodlands (White 1983). The Zambezi Mopane region is characterized by the tree flora being dominated by the Mopane tree (*Colophospermum mopane*). This species of tree was originally identified as potential reservoir of *C. neoformans* by Litvintseva *et al*. (2006) and covers 15% of Zambia, entering the country from the Botswana border. The Zambezian Mopane woodlands follow a low‐elevation area ranging from 200 to 600 m as opposed to the Central Miombo Woodlands which are contained between 800 and 1200 m. The Zambezi Mopane Woodlands region is thought to have significant evolutionary implications for biodiversity as it represents a changeover zone between tropical and subtropical biomes (World Wildlife Fund 2011), and remains a centre for Zambia's National Game Parks, chiefly the Luangwa valley. Covering more than 50% of the country, the Central Miombo woodlands represent the most extensive ecoregion in Zambia. The dominant tree type is *Brachystegia* sp.*,* but sporadic grassy wetlands (‘dambos’) can make up to 30% of this ecoregion. The population density in the region is relatively low principally owing to the low‐productivity agriculture caused by nutriment‐poor soils that are dominated by Kalahari sands (World Wildlife Fund 2011). Subsistence slash‐and‐burn (chitemene) agriculture is practiced by a large proportion of the population, and as a consequence, the Miombo woodlands have been extensively deforested in the recent years.

Samples were collected in using Transwab^®^ Amies swabs (MWE^TM^ – MW170) and sterilized 30‐mL screw‐capped glass bottle. Amies liquid transport swabs were taken from tree bark. Pairs of samples from bark and soil associated with each tree were also collected. Samples were collected and processed according to previously established protocols (Randhawa *et al*. 2005; Litvintseva *et al*. 2011), and the samples were kept at 4 °C until been processed on niger seed agar. All samples were collected under licence from the Zambian Wildlife Authority (ZAWA).

### Isolation and identification of *C. neoformans* and *Cryptococcus gattii*

Niger seed medium is broadly used to identify both *C. gattii* and *C. neoformans* isolates as they produce melanin on the medium and can be distinguished from other brown yeast colonies from their characteristic dark brown colour (Staib 1987). Niger seed plates were incubated at 30 °C for 48 h. To obtain the niger seed medium, 70 g of niger seeds (*Guizotia abyssinica*) was pulverized and added to 1 L of distilled water. The mixture was autoclaved for 15 min. After allowing the solution to cool off, the solution was then filtered through using a triple layer of cheesecloth. One gram of glucose (Sigma‐Aldrich), 1 g of KH_2_PO_4_ (Sigma‐Aldrich), 0.78 g of creatinine (Sigma‐Aldrich) and 15 g of agar (Sigma‐Aldrich) were added to the niger seed extract. Distilled water was added accordingly to obtain 1 L of niger seed extract solution, and the medium was autoclaved a second time for 30 min. Before dispensing the mixture onto culture plates, 0.05 g of chloramphenicol (Sigma‐Aldrich) was resuspended in 1 mL of 95% ethanol and added to the niger seed medium to inhibit bacterial growth. The tubes containing soil or animal droppings were weighted to 0.5 g and distilled in 10 mL of distilled water. The samples were then further diluted to 1/10 and 1/100. Swabs were suspended in 1 mL of distilled water and diluted to 1/10. Then, 80 μL of both the undiluted and diluted samples was spread onto niger seed plates and each dilution was duplicated. Single colonies were isolated from plates and subcultured. During genomic DNA extraction, all purified single colonies were cultured for 60 h at 37 °C in a 50‐mL falcon tube containing in 5 mL of yeast protein digest (YPD) media supplemented with 0.5 m of NaCl at 250 rpm in order to reduce capsule size. The extraction process was performed using MasterPure Yeast DNA purification kit (Epicentre, UK) using a bead‐beating step with Mini‐Beadbeater‐16 (Cat. No. 607; Biospec). The genomic DNA was amplified by polymerase chain reaction (PCR) to distinguish between *C. gattii* and *C. neoformans* (*Cn*) using two sets of primers: *CAP59*, a capsular gene present in both *Cn* and *C. gattii* species, and *SOD1*, which is specific for *Cn* (Meyer *et al*. 2009).

To avoid contamination with filamentous fungi, individual yeast colony resembling *C. neoformans* (dark yellow/brown colonies) was isolated further onto another niger seed agar plate and on SDS agar. For each sample, a total of five individual dark brown colonies were isolated and their DNA was extracted. The extraction process was performed using MasterPure Yeast DNA purification kit (Epicentre) using an additional bead‐beating step. The genomic DNA was amplified by PCR to distinguish between *C. gattii* and *C. neoformans* using two sets of primers: *CAP59*, a capsular gene present in both *Cryptococcus neoformans* and *C. gattii* species, and *SOD1*, which is specific for *C. neoformans* (Meyer *et al*. 2009). A Pearson's chi‐squared test was used to assess potential differences in the proportions of *C. gattii* and *Cn* in the different ecoregions investigated. The test statistic was implemented in r software (v 3.1.1).

### Mating types

The mating type for each environmental *Cn* isolates was determined using PCR analysis. Reactions were performed in a total volume of 25 μL and comprised of 0.8 μL of each of the following primers (10 μm), JOHE7264, JOHE7265, JOHE7270, JOHE7272 (de Oliveira *et al*. 2004). 0.5 μL of MgCl_2_ was added to the reaction mix with 0.5 of dNTPs (10 μm) (R0191; ThermoFisher), 2.5 μL of Rxn Buffer, 17.4 μL of H_2_O and 0.1 of Taq polymerase (cat: 18038018; ThermoFisher). Reaction conditions were as follows: *JOHE*7264/*JOHE*7265; *JOHE*7072/*JOHE*7272‐: Control method: calculated: (i) initial denaturation (94 °C for 3:00 min), (ii) denaturation (94 °C for 30 s), (iii) annealing (55 °C for 30 s), extension (72 °C for 1:00 min), (iv) go to (ii), (× 35), (v) final elongation (72 °C for 10:00 min) and 4 °C for infinity.

### Fungal community structure using an *ITS2* metabarcoding

The ITS region provides an ideal target to detect and delineate fungal species and has been extensively used for the taxonomic profiling of fungi (Xu 2006; Lindahl *et al*. 2013). In 2012, this genetic marker was proposed as the universal genetic barcode for fungi (Schoch *et al*. 2012). A comparison between *ITS1* and *ITS2* revealed that *ITS2* was more variable and allowed a better assessment of the fungal diversity (Bazzicalupo *et al*. 2013). Therefore, the current study used *ITS2* for metabarcoding sequencing. The *ITS2* rDNA region was amplified using ITS3 KYO2 and ITS4 KYO3 (Toju *et al*. 2012). The two primers were paired with appropriate Illumina adapter overhang nucleotide sequences and a 2‐bp linker sequence. The *ITS2* region of 61 samples taken from soil adjacent to sampled trees was amplified using an Illumina MiSeq instrument using a 300‐bp paired‐end sequencing run. Eleven samples which were found positive for the *Cryptococcus* species complex were included in the sample panel to assess whether the particular mycobiome was associated with the presence of *Cryptococcus*. Briefly, 0.25 g of soil was weighted and transferred to PowerBead Tubes (MO BIO PowerSoil DNA Isolation Kit). Amplifications were carried out in at total volume of 25 μL using 2.5 μL (5 ng/μL) of DNA, 12.5 μl 2× KAPA HiFi HotStart Ready Mix (ANACHEM, catalogue: KK2602) and 5 μL (1 μm) of each primer. PCR conditions for ITS3_KYO2/ITS4_KOY3: Control method – calculated: (i) initial denaturation (94 °C for 3:00 min), (ii) denaturation (94 °C for 30 s), (iii) annealing (55 °C for 30 s), extension (72 °C for 1:00 min), (iv) go to (ii), (× 25), (v) final elongation (72 °C for 5:00 min). Products were then kept at −20 °C until DNA precipitation step. Amplicons were visualized using Agilent 2200 TapeStation (Agilent Technologies, Inc). The libraries were then prepared according to a standard Illumina Protocol. Soil constitutes a highly complex environment that is mostly unexplored in sub‐Saharan Africa, and a better understanding of its structure and diversity is required to understand the fungal interaction with other organisms (Lim *et al*. 2010). For each sample, between 5 and 10 g of soil were collected in various ecoregions in Zambia during both the dry and rainy seasons. Soil was kept at 4 °C during field collection and frozen at −20 °C in the laboratory until being processed. DNA extraction and PCR amplification of the genomic DNA were extracted from 74 soil samples collected in various part of Zambia using the PowerSoil DNA Isolation Kit (cat. 12888‐100; MOBIO Laboratories, Inc.). A negative control was used to account for eventual taxa resulting from laboratory contamination. The genomic DNA was then purified and Illumina paired‐end libraries were prepared according to the Illumina protocol. The *ITS2* region was amplified using an Illumina MiSeq instrument on a 300‐bp paired‐end sequencing run. Paired‐end libraries were prepared for sequencing in a two‐step PCR approach using the Illumina 16S Metagenomic Sequencing Library Preparation Protocol. The *ITS2* rDNA region was amplified using *ITS3_KYO2* and *ITS4_KYO3* (Toju *et al*. 2012). The two primers were paired with appropriate Illumina adapter overhang nucleotide sequences and a 2‐bp linker sequence. The sequence of the two primers with linker and overhang sequences are detailed on Fig. S1.

### Environmental variables

A number of environmental data were incorporated into the analysis to explore the relationship between environmental factors and the *Cryptococcus* species complex. Data were selected aiming to measure the operational environment of the saprophyte. Other studies which aimed to model the fundamental niche of the pathogen identified relevant climatic and spatial variables to study the distribution of *Cryptococcus* using a jackknifing procedure (Mak 2007; Mak *et al*. 2015). In the present study, the same environmental variables were explored. *Cryptococcus* is known to be sensitive to temperature and atmospheric conditions, and these variables are investigated using the WorldClim Global Climate Data (Busby 1991). Additionally, altitudinal influence, Normalized Difference Vegetation Index (NDVI) and Enhanced Vegetation Index (EVI) were also considered as they are commonly used as predicators in disease mapping (O'Hanlon *et al*. 2016). Environmental variables were extracted from WorldClim Global Climate Data (Busby 1991), and the soil information was found using the Africa Soil Information Service (Hengl *et al*. 2015). The information for each sample was extracted in r software (v 3.1.1) using the raster (Hijmans *et al*. 2012) and dismo (Hijmans *et al*. 2012) packages. The EVI and the NDVI from 2012 and 2014 were extracted and averaged over this period using MODISTools (Tuck *et al*. 2014).

### Ecological niche modelling

Zambia provides an appropriate setting to model the ecological niches of *C. neoformans* and *C. gattii*. maxent is an species habitat modelling software, and it uses maximum entropy to model species’ geographic distribution (Phillips & Dudík 2008) based on presence‐only data. The software was used to generate the predicted distribution of the two sister species in Zambia. The distribution was modelled using Bioclim layers (Busby 1991) and altitude. The niche model produced by maxent is an approximation of the species’ ecological niche within the context of the environmental dimensions that we investigated. The purpose of the software advanced by Phillips *et al*. (2006) is to make predictions from incomplete information. maxent calculates the probability distribution of the maximum entropy. The latitude and longitude of the isolates constitute the sample points and the Bioclim layers the environmental features. To test model prediction, 25% of the samples were randomly set aside (Fig. S9, Supporting information).

### Whole‐genome sequencing and SNP calling

High molecular weight DNA was extracted, and DNA libraries were prepared for Illumina HiSeq paired‐end sequencing. Our studies focused on *C. neoformans* as this pathogen represents the majority of cryptococcal meningitis cases in Zambia and, more widely, southern Africa. Environmental isolates collected from the environment were sequenced along with 23 clinical *C. neoformans* genomics acquired from an ACTA Lusaka Trial in Lusaka, Zambia. *C. neoformans* colonies were cultured in 5 mL of YPD media (Sigma‐Aldrich) supplemented with 0.5 m of NaCl for 60 h at 37 °C, shaking at 165 rpm. Forty‐seven environmental and clinical genomes from single‐colony isolates of *Cryptococcus* collected across Zambia were sequenced with average 100× coverage. DNA concentrations were measured with a Qubit^®^ Broad Range dsDNA Assay (Life Technologies™) on Qubit^®^ 2.0 Fluorometer (Q32866; Invitrogen™) according to the manufacturer's instructions. The genomic DNA was then diluted to 2 ng/μL in nuclease‐free water. Libraries were prepared using the TruSeq^®^ Nano DNA Sample Preparation Kit (Illumina; FC‐121‐4001). Each time, 24 libraries were prepared for paired‐end sequencing on two lanes of Illumina^®^ HiSeq to sequence 175‐bp fragments. Libraries were quantified by qPCR on an Applied Biosystems 7300 instrument (Life Technologies) using the Kapa library quantification kit (Kapa Biosciences, Boston, MA, USA) and the 2200 TapeStation (Agilent) with D100K ScreenTape assays (Agilent) as described in Rhodes *et al*. (2014). The information of these two assays was subsequently used to determine the dilution needed for normalizing each library to the same concentration and the pooling of each library. The libraries were then normalized to 10 nm and pooled together (12 samples/pool). The pooled libraries were sequenced by the Medical Research Council Clinical Genomics Centre (Hammersmith, London, UK) to a read length of 100 bp on an Illumina^®^ HiSeq 2000 or 2500 sequencer. A total of 12 isolates were sequenced per lane of each flow cell, loading 16 pm of the pooled libraries. All raw reads and lineages information regarding each isolates have been submitted to the European Nucleotide Archive (MiSeq ITS project: PRJEB13820; WGS *C. neoformans* Project: PRJEB13814).

Reads were aligned to the *Cryptococcus neoformans* H99 reference (Loftus *et al*. 2005) using the short‐read alignment component of Burrows‐Wheeler Aligner (BWA) 0.75a aln (Li & Durbin 2009) using a quality threshold of 15 as described by Rhodes *et al*. (2014). FastQs were converted to SAM format using BWA and converted to BAM files, and the BAM files were then sorted and indexed with samtools version 0.1.18 (Li *et al*. 2009). Duplicated reads were marked with picard tools (v. 1.72; github.com/broadinstitute/picard). The resulting BAM files were recalibrated around insertions or deletions (INDELs) using the GATK RealignerTargetCreator and IndelRealigner (McKenna *et al*. 2010). The detection of single nucleotide polymorphisms (SNPs) and INDELs was called using GATK unifiedgenotyper version 2.2‐2 in haploid mode (DePristo *et al*. 2011; Van der Auwera *et al*. 2013). SNPs and INDELs were filtered to call only high‐confidence variants, according to whether they were present in 80% of reads. Mapped reads for each isolates are given on Table S9 (Supporting information).

### Phylogeny and population assignment

Whole‐genome SNPs files were converted to Nexus and Phylip format. raxml, executing 1000 repaid bootstrap inferences with a generalized time reversible substitution matrix (Stamatakis 2006) was used to generate bootstrapped maximum‐likelihood trees over 1000 replicates, which were visualized in figtree version 1.4.0 (http://tree.bio.ed.ac.uk/software/figtree/). tempest v1.5 was used to root the phylogenetic tree (Fig. 2) and to ensure that a strict molecular clock could be applied (Rambaut *et al*. 2016). To first analyse the population structure of environmental *Cryptococcus* populations, we used ChromoPainter and *fineStructure* (Lawson *et al*. 2012). ChromoPainter constructs a coancestry matrix based on individual SNPs. Each individual is considered in terms of either being a donor or a recipient of ‘chunks’ of DNA. The coancestry matrix then records the inferred recombination events between each donor and recipient prior to coalescing with another genome.

### Statistical analysis

##### ITS2 metabarcoding

Raw Illumina fastq files were merged into paired‐end reads using PANDASeq (Masella *et al*. 2012). Then, the clustering of ITS2 sequences, their taxonomic assignments and the analyses were performed using the quantitative insights into microbial ecology (qiime) software v1.8.0 (Caporaso *et al*. 2010). A chimera filtering step was performed using usearch v7 (Edgar 2010). The clustering and assignment of reads into operational taxonomic units (OTUs) were achieved using the OTUs‐picking workflow in qiime, and reads were grouped using usearch and uclust. These algorithms divide sequences into clusters using a 97% threshold of pairwise identity and a maximum *e*‐value of 0.001 (Edgar 2010). The OTU table was rarefied at a sequencing depth of 300 to remove sample heterogeneity. The UNITE fungal its database (v.12.9) (Abarenkov *et al*. 2010) was used to map OTUs to a reference and the identification of extracted *ITS2* OTUs was performed using blast. A negative control was included and reads from the negative control were removed; these were assigned at the genus level to *Trichosporon* and others to ‘unidentified fungi’. α− and β‐diversity were calculated using qiime; α‐diversity is known as the species richness of a particular community (Whittaker 1960) and β‐diversity is the extent of change of community composition across space and time. Rarefaction curves are obtained from α‐diversity measures (observed OTUs and chaos1 estimate) and allowed to assess taxonomic richness of the samples (Magurran 2013). β‐Diversity was estimated using the Bray–Curtis metric. This method allows to calculate pairwise distances between fungal communities. Principal coordinate analysis were computed using the core‐analysis framework within qiime. To investigate potential differences in microbial β‐diversity between ecoregions or seasons, the analysis of similarity test (ANOSIM) and ADONIS with 999 permutations were used between the different categories based on Bray–Curtis dissimilarity.

ANOSIM is based on a standardized rank correlation analysis between two matrices. The test is commonly used in community ecology. ANOSIM examined the variation in species composition among different grouping factors. Samples are assigned to groups and ANOSIM test whether there are significant differences between these groups (Clarke 1993). A *P*‐value of 0.001 indicates significant differences between the groups at α = 0.05. An R coefficient superior to 0.25 implies that groups are different with some overlap. If the R coefficient exceeds 0.5, the groups are considered different (Fierer *et al*. 2010). ADONIS is similar to ANOSIM and is widely used in analysis of ecological community data. It is common practice in community ecology to combine these two statistics. The ADONIS method assesses the significance between sample groups based on a distance matrix. The analysis is similar to anova. The statistical significance is achieved by partitioning the sum of squares of the data set based on permutations and using Bray–Curtis matrices. Then, the method computes a *R* ^2^ coefficient which transcribes the percentage of variation explained by the categories and gives the statistical significance (Anderson 2001).

One‐way analysis of variance (anova) was used to identify taxa which differed between sample groups. A best variables rank correlation test (BEST) was then performed to rank the relative importance of environmental conditions on β‐diversity. The BEST analysis identifies subsets of variables whose Euclidian distances are maximally correlated with the Bray–Curtis matrix. The correlation is computed using a Spearman's rank correlation coefficient (Spearman 1904). Multivariate analysis of variance (manova) also known as ‘permutation anova’ was performed within the two main ecoregions (Miombo woodland and Zambezi Mopane) with 999 permutations. This method is similar to ADONIS as it partitions sums of squares. The method was used to assess the influences of the climatic factors within each ecoregion by returning a pseudo‐*F* value and a *P*‐value. Only OTUs present in more than 0.001% of the total filtered sequences were considered when comparing ecoregions and seasons. To highlight the taxa overlap within the different ecoregions, Venn diagrams were generated based on OTUs presence and visualized using http://bioinfogp.cnb.csic.es/tools/venny/index.html Oliveros (2007–2015). Taxonomic differences between samples of each ecoregion were tested using linear discriminant analysis (LDA) effect size (Segata *et al*. 2011). We first employed the factorial Kruskal–Wallis sum‐rank test (α = 0.05) to identify taxa with significant differential abundances between categories (ecoregion and season). Then, LDA effect size was applied to estimate the effect size of each differentially abundant taxa.

##### Population genetics

General population statistics were generated for VNI and VNB lineages of *C. neoformans*; the number of segregating sites (S), total number of mutations, number of singletons, nucleotide diversity (π), Waterson's estimator (θ), and Tajima's *D* and LD′ were estimated using variscan v.2.0 (Hutter *et al*. 2006) (Table S13, Supporting information). The nucleotide diversity measures the degree of polymorphism in a population (Nei & Li 1979). The Waterson estimator estimates the population mutation rate and decreases when the sample size or the recombination rate increase (Watterson 1975).

## Results

### Recovery of *Cryptococcus* in Zambia

We sampled the two main ecoregions of Zambia: Central Miombo woodlands (MW; *n *=* *314 sites) and Zambezi Mopane Woodlands (ZM; *n *=* *304 sites) (Table 1). A total of 1391 samples were collected from soil and trees across these Zambian ecoregions during both the dry (*n *=* *773) and rainy (*n *=* *618) seasons (Fig. S2, Supporting information). A total of 24 isolates from geographically distinct samples for *Cryptococcus neoformans* and 38 *Cryptococcus gattii* were identified. The species recovery rate of the *Cryptococcus* species complex was 5.2% in the rainy season and 3.9% in the dry season. The Zambezi Mopane ecoregion was most strongly associated with *C. neoformans* (19/24 isolates), whereas *C. gattii* was more strongly associated with the Central Miombo ecoregion (32/38 isolates); each of these species was significantly associated with their respective ecoregion (*P *<* *0.001, Pearson's chi‐squared test).

**Table 1:** Abbreviations

Abbreviation	Name
*Cn*	*Cryptococcus neoformans*
*Cg*	*Cryptococcus gattii*
ZM	Zambezi Mopane Woodlands
MW	Central Miombo woodlands
WGS	Whole‐genome sequencing
MLST	Multilocus sequence typing
YPD	Yeast protein digest
QIIME	Quantitative insights into microbial ecology
OTUs	Operational taxonomic units
PCoA	Principal coordinate analysis
ANOSIM	Analysis of similarity test
anova	Analysis of variance
manova	Multivariate analysis of variance
BEST	Best variables rank correlation test
LDA	Linear discriminant analysis

### Association between environmental factors and fungal community structure

**Figure 1:**
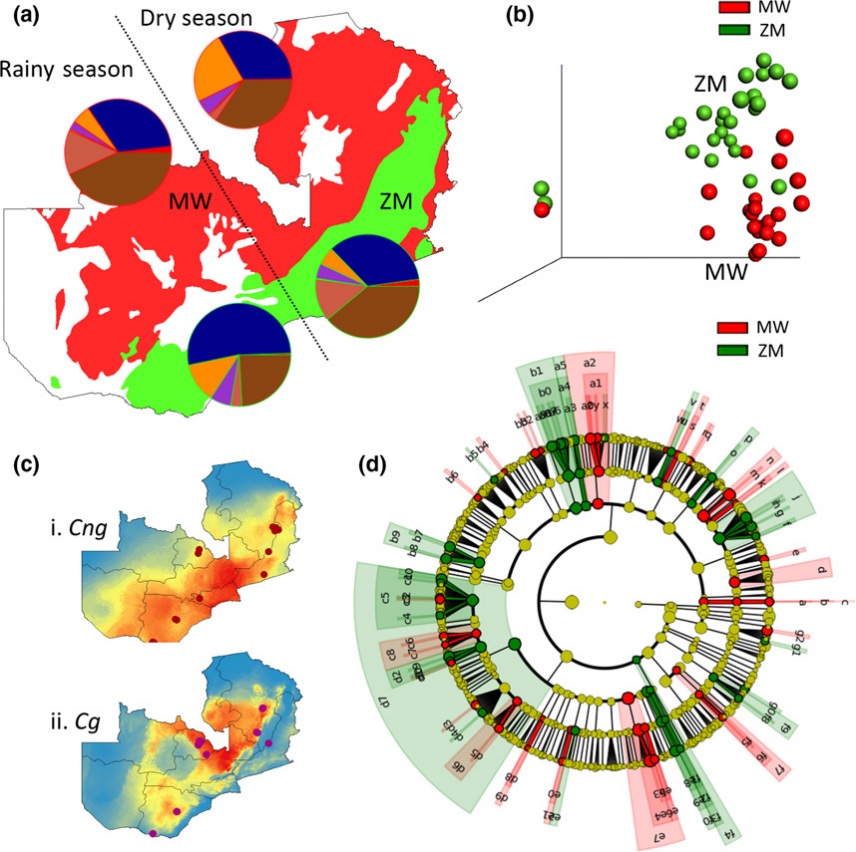
(a) Variation of the *Cryptococcus*‐associated fungal community structure across the two main Zambian ecoregions during the dry and rainy seasons among the Zambezi Woodlands (ZM; green) and Miombo Woodlands (MW; red). Pie charts represent the profile of fungal phylafor each season: Ascomycota (blue), Basidiomycota (orange), Chytridiomycota (purple), Glomeromycota (yellow), Zygomycota (coral), other (red) and unidentified fungi (brown). (b) PCoA plots of fungal diversity for MW (red) and ZM (green) illustrating the difference in mycobiome across each ecoregion. (c) Environmental niche modelling for the two sister species showing the predicted distribution of *C. neoformans* (*Cn*) (i) is biased to the ZM ecoregion, whereas *C. gattii* (*Cg*) (ii) is biased to the MW. Each dot represents a positive sample for either species. (d) LDA effect size taxonomic cladogram comparing fungal community categorized by ecoregion and season. Branch areas are shaded according to the highest ranked variety for that taxon, and the significantly discriminant taxon nodes are coloured in green (ZM) or red (MW). Nonsignificant taxon appears in yellow. Highly abundant and select taxa are as follows: d9, Tremellales; b1, Pezizomycetes; a2, Leotiomycetes; j, Pleosporales. The complete list of discriminate taxa and ranks are listed on Fig. S7 (Supporting information). [Colour figure can be viewed at wileyonlinelibrary.com]

Modelling the ecological niches of the two sister species across Zambia aimed to predict the realized niche of *C. neoformans* and *C. gattii*. The predictive environmental niche model built using spatial and climatic variables (Fig. 1) showed that the ecological niche of *C. neoformans* is largely associated with the *C. mopane* tree belt, whereas the realized niche of *C. gattii* is largely within the wetter, higher altitude, Central Miombo woodlands. The environmental variables which gave the highest relative contribution to the model were for *C. neoformans*: Isothermality (bio3) accounting for 71.2% of the model variation and Precipitation Seasonality (bio15) accounting for 22.0%. With respect to *C. gattii*, the relevant climatic variables were Isothermality (bio3) 26.5%, ‘Precipitation of Wettest Quarter’ (bio16) 18.4%, ‘Precipitation of Warmest Quarter’ (bio18) 16.8% and ‘Mean Temperature of Driest Quarter’ (bio9) 7.9% (Table S1, Supporting information).

Fungal community structure assessed through high‐throughput barcoding was shown to vary significantly across the two ecoregions investigated (*R*_ANOSIM_ **=* *0.3704, *P *<* *0.001) (Table 2). Elevation, latitude and longitude improved regression coefficients showing that these factors are playing a significant role in the distribution of fungal communities where the *Cryptococcus* genus is embedded (Table S3, Supporting information). These variables were also significant when investigated using ADONIS, excepting for longitude (*R* ^2^_ADONIS_ **=* *0.0183, *P *<* *0.198). The microbial compositions of the 11 positive samples were compared to nonpositive sites (*n* = 50) in an attempt to identify potential taxa associated with either *C. gattii* or *C. neoformans*. However, no significant associations could be detected (*R*_ANOSIM_ **=* *0.0633, *P *<* *0.263). We found that the abundance of fungal phyla varied between each ecoregion. Generally, the number of sequences per sample was higher in Zambezi Mopane Woodlands samples (Zambezi Mopane* *=* *207 OTUs per sample, Miombo woodland* *=* *149 OTUs per sample, *P *<* *0.001). Across the Zambezi Mopane woodlands, Ascomycota represented 35.61% (*n*_OTUs_ **=* *3356) of all OTUs, followed by Basidiomycota (11.15%, *n*_OTUs_ **=* *1754). Across the Central Miombo woodlands samples, Ascomycota represented 32.69% (*n*_OTUs_ **=* *2392) and Basidiomycota 11.20% (*n*_OTUs_ **=* *1740). Figure 1a portrays the fungal community structure associated with each ecoregion and the complete distribution of OTUs among samples is given on Figs S4–S6 (Supporting information). A total of 1866 genera were identified and were found to be unevenly distributed across each ecoregion (Miombo woodland* *=* *754, Zambezi Mopane* *=* *1112). The fungal diversity across each of the different ecoregions investigated was compared (Fig. S3a, Supporting information), and rarefaction curves based on Chaos1 α‐diversity metric showed a saturation within the two ecoregions (Figs S3b and S6, Supporting information). This indicates that the OTUs identified in the survey accounted for a large proportion of the expected fungal diversity present in Zambian soil. Our analysis investigated samples over 900 km apart within Zambezi Mopane Woodlands and 400 km within Central Miombo woodlands, and no significant variation could be observed (Zambezi Mopane: *R*ANOSIM* *=* *−0.008, *P *=* *0.499; Miombo woodland: *R*ANOSIM* *=* *0.106, *P *=* *0.189), indicating that soil fungal diversity appeared relatively uniform across space within each ecoregion. On one hand, season played a significant role in shaping the microbial diversity in the Zambezi Mopane ecoregion (*R*_ANOSIM_ **=* *0.3610, *P *=* *0.019) while, on the other hand, the patterns of fungal diversity in the Central Miombo woodlands appear to remain stable across seasons (*R*_ANOSIM_ **=* *0.1062, *P *=* *0.189) (Table S4, Supporting information). Linear discriminant analysis effective size revealed broad taxonomic trends in the mycobiome associated with the two ecoregions with the Tremellales (the order of wood‐rotting jelly fungi of which *Cryptococcus* is a member) being more abundant in the Zambezi Mopane Woodlands (Figs 1d, S7 and S8, Supporting information).

**Table 2:** ANOSIM and ADONIS of microbial diversity patterns within Zambian Ecoregions

		Bray–Curtis dissimilarity			
		ANOSIM		ADONIS	
Group	Factor	*R*	*P*	*R* ^2^	*P*
Central Miombo woodlands	Ecoregions	0.4184	0.001	0.0428	0.001
Zambezi Mopane Woodlands	Ecoregions	0.2726	0.001	0.0416	0.001
Zambezi Mopane Woodlands	Central Miombo woodlands	0.3704	0.001	0.0687	0.001

### Whole‐genome sequencing

**Figure 2:**
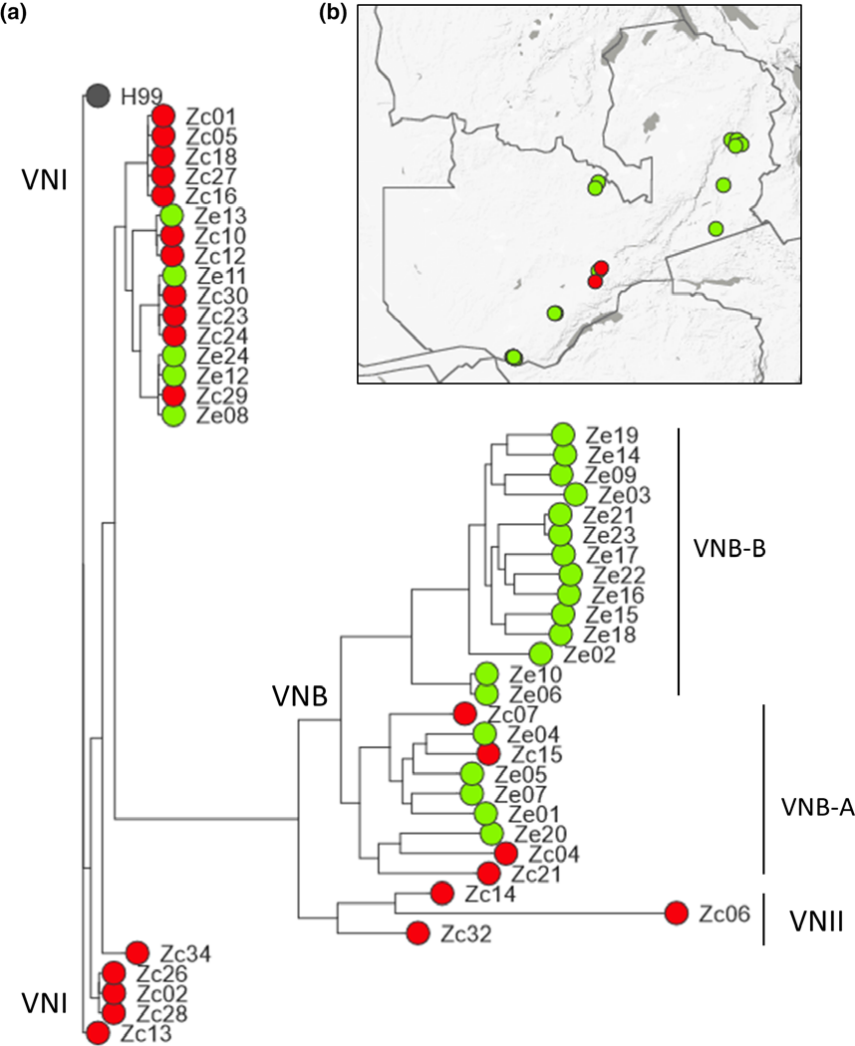
Phylogenetic relationship between environmental and clinical isolates of *C. neoformans* compared against the H99 reference detailing association with the known lineages of *C. neoformans*. (a) Clinical isolates appear in red, whereas environmental isolates are in green. Phylogenetic analysis were performed using maximum‐likelihood‐based inference (raxml) (Stamatakis 2006) using SNP data; all branches had bootstrap values of 100 with 1000 generations. The tree was rooted using TempEst. Isolate references include two letters, Z – Zambia and either clinical – c, or environmental – e; (b) Distribution of the environmental or clinical *C. neoformans* isolates collected and sequenced throughout Zambia. The phylogenetic representation here was generated within a Microreact Project https://microreact.org/project/S1lkajtY. [Colour figure can be viewed at wileyonlinelibrary.com]

**Figure 3:**
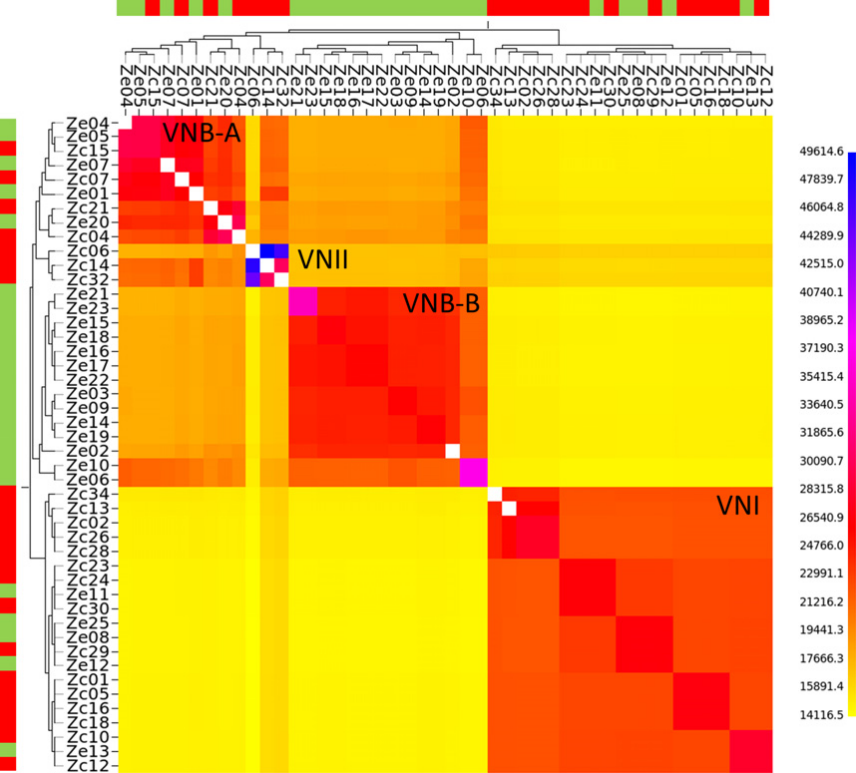
*FineStructure* analysis of *C. neoformans* population structure in Zambia for clinical (red) and environmental (green) isolates. On the *x*‐axis, each genome is considered as a recipient, and on the *y*‐axis, the *C. neoformans* isolate is considered a donor of genomic region. The VNB and VNI populations are clearly separated with very limited sharing of genomic regions between the VNI and VNB populations. The highest amount of shared genome regions between isolates appears in purple and the lowest in yellow. [Colour figure can be viewed at wileyonlinelibrary.com]

For our phylogenetic analyses, owing to the predominance of pathogenic isolates being *C. neoformans*, we focused our attention on this species. Mapping Illumina reads (average reads mapped per isolate* *=* *88%) for the 24 environmental and 23 clinical Zambian *C. neoformans* against the 19 Mb VNI H99 reference genome (Loftus *et al*. 2005) identified a total of 822 772 SNPs (Table S9, Supporting information). On average, reads were mapped at 88% to the reference. Phylogenetic analysis determined the occurrence of 21 VNI, 23 VNB and 3 VNII *C. neoformans* in our panel (Fig. 2). These data can be visualized in a Microreact project at https://microreact.org/project/S1lkajtY. The majority of the clinical isolates were VNI isolates (76%) showing that this molecular type dominates in human infections. Strikingly however, the large majority of environmental isolates (83%) belonged to lineage VNB. As shown in Fig. 2, the VNI, VNII and VNB molecular types clearly constitute separated lineages, supporting earlier work from southern Africa using multilocus sequence typing (MLST; Litvintseva *et al*. 2003, 2006, 2011; Simwami *et al*. 2011; Beale *et al*. 2015; Chen *et al*. 2015). These results were corroborated with *fineStructure* analysis (Fig. 3), where very limited genetic exchange was observed between the different molecular types. Among VNI (*n *=* *24) and VNII isolates (*n *=* *3), 105 391 and 366 086 SNPs were found, respectively. The VNB clade harboured much higher genetic diversity with a total of 521 924 SNPs being mapped (Table S10, Supporting information). Within VNB, two clearly defined subpopulations, VNB‐A and VNB‐B, were observed to subdivide this highly genetically diverse lineage (Figs 2 and 3). VNB‐A and VNB‐B were found to have 228 688 SNPs in common; 170 258 SNPs were private to VNB‐A and 188 273 were private to VNB‐B. All 14 isolates of VNB‐B were environmental in their origin, whereas four of nine isolates of VNB‐A were recovered from HIV‐AIDS patients.

Population genomics of environmental VNB isolates showed that these environmental isolates group into two statistically supported clades with 35% of VNB isolates possessing the *MATa* mating‐type locus. VNI isolates displayed a more clonal profile with the *MAT*a mating‐type being much rarer (4%). VNI infections likely represent urban acquired infections, and VNB *C. neoformans* which are mainly present across the *Colophospermum mopane* region, likely reflect infection acquired in rural settings. Genetic diversity is higher in the VNB clade with the number of segregating sides (S), number of mutations (η), number of external mutations (ηE), number of nucleotide differences per site (π) greatly exceeding those found in VNI (Table S13, Supporting information). Among the VNB isolates, nine of the 23 isolates possessed the *MAT*a locus (39%). Within the *fineStructure* analysis (Fig. 3), two VNB subpopulations could be observed, VNB‐A and VNB‐B. In the VN VNB‐A clade, five were positive for *MAT*a, and six were positive for *MAT*α locus (45% *MAT*a). In the VNB‐B lineage, three isolates were *MAT*a positive and seven possessed *MAT*α (30% *MAT*a). In the VNI clade, only one of the 21 isolates possessed the *MAT*a locus (4%). The mating type of each isolate is listed on Table S9 (Supporting information). The VNB molecular type displayed a slightly negative Tajima's *D* value (Tajima's *D* = −0.265) indicating a surplus of rare alleles, whereas the VNI population had a strong positive value (Tajima's *D* = 0.884), indicating a sudden population contraction or balancing selection (Tajima 1989) (Table S13, Supporting information).

## Discussion

We report here the first population genomic analysis of *Cryptococcus neoformans*, a fungus that is responsible for hundreds of thousands of deaths each year in Africa and which accounts for around 17% of AIDS‐associated mortality. Our use of high‐resolution WGS to analyse environmental and clinical populations of this pathogen has uncovered hitherto unmapped levels of genomic diversity, and we show that this diversity is deeply structured across even the scale of the single African country, Zambia, within which our study took place. These data have allowed the identification of three lineages of *C. neoformans* that occur within Zambia, describing highly divergent epidemiological patterns that reflect the environmental reservoirs of infection that our study defines.

Our genomic data extend the findings of Chen *et al*. (2015) who showed that the VNB molecular type is widely present in the arboreal environment, specifically the Mopane belt, across the neighbouring country of Botswana. The high recovery of VNB from Mopane trees across Zambia confirms the Zambezi Mopane Woodland ecoregion as the main niche for *C. neoformans* VNB. Strikingly, our phylogenomic analysis showed that VNB is not only over fivefold more genetically diverse than the pan‐global lineage VNI, but also contained two strongly supported subclades, here named VNB‐A and VNB‐B. A previous report by our group in South Africa showed that patients infected with the VNB molecular type had significantly worse survival than those infected with VNI (Beale *et al*. 2015). To which subclade of VNB the South African virulent clinical isolates belong is not currently known, as is the relative virulence of Zambian clinical VNB‐A compared to their environmental VNB‐B or clinical VNI counterparts. These analyses await the cessation of the ACTA Lusaka Trial and subsequent analysis of the trial data in conjunction with a larger panel of sequenced isolates from across the full longitudinal spectrum of samples recovered from Zambian patients.

Over three‐quarters of clinical infections in our Zambian cohort were caused by the VNI molecular type. We found a similar pattern to that described by Chen *et al*. (2015) in Botswana, where VNI was associated with clinical infections in urban areas and VNB was found strongly associated with Mopane trees in rural settings. In both studies, the VNII molecular type was found to be rare. Although it is not possible to infer where patients in the trial acquired their infections due to the potential for reactivation of latent infection in the setting of profound immunosuppression, taken together our studies support the hypothesis that intensified urbanization across southern Africa has led to high densities of domestic pigeons, *Columba livia*, which have led to an amplification of VNI‐related infections in cities such as Lusaka and Gaborone (Litvintseva *et al*. 2011; Simwami *et al*. 2011). We find that 96% of VNI isolates are the *MAT*α mating type, a finding that highlights the clonal profile of the VNI molecular type compared to VNB isolates which displayed a high proportion (36%) of the *MAT*a mating type. The current understanding of the evolutionary history of VNI derived from MLST analyses is that the low‐diversity, pan‐global, nature of its genotype reflects a global dissemination alongside the domestication of pigeons within recent history (Litvintseva *et al*. 2011; Simwami *et al*. 2011). Our genomic data support this theory by confirming that VNI is genetically depauperate compared to both VNII and VNB. Further understanding of the evolutionary epidemiology of these lineages within their global context awaits the broad‐scale phylogenomic studies that will inevitably follow our studies lead.

Across the broader species complex, we show that *C. neoformans* and its sister species, *C. gattii*, were associated with different ecotypes in Zambia. This shows that the evolutionary separation of these two species, which speciated around 37 Ma (Xu *et al*. 2000), has resulted in their adaptation to occupy markedly different ecological niches. *C. neoformans* was predominantly recovered from trees, mainly *Colophospermum mopane* (49% of sampled trees) that dominated the Zambezi Mopane ecoregion. In comparison, *C. gattii* was found predominately in the Central Miombo Woodlands, where 32 of the 38 isolates (84%) were isolated. The dominant tree type in the Miombo woodlands is *Brachystegia* sp.*,* and six *C. gattii* isolates (16%) were recovered from these trees (Table S12, Supporting information). Two isolates of *C. gattii* were also found on *Eucalyptus*, a tree species which has been associated with *C. gattii* for decades (Ellis & Pfeiffer 1990; Pfeiffer & Ellis 1992). Interestingly, we also found that *C. gattii* was strongly associated with hyrax faeces (15 isolates in 52 samples). This finding identifies a new potential animal reservoir for *C. gattii* and emphasizes how ubiquitous this pathogen is. Additional sampling will lead to better understand the evolutionary history of the *Cryptococcus* species complex in Africa, especially in relation to their association with potential vertebrate hosts.

We used ecological niche modelling to further study the abiotic niche requirements for *C. gattii* and *C. neoformans*. These analyses confirmed the influence of climate on the distribution of the two species by predicting their environmental tolerances (Kearney & Porter 2004). Projections by maxent closely matched the two main ecoregions; the Central Miombo woodlands for *C. gattii* and the ZM for *C. neoformans*. Relevant climatic variables explaining *C. neoformans* distribution under our maxent model were also identified in our manova (Table S5, Supporting information) and best variables rank correlation test (BEST) approach (Appendix S1) with isothermality and precipitation seasonality accounting for most of the model variation, describing 71% and 22% of *C. neoformans* distribution, respectively. Precipitation clearly plays a significant role in predicting the distribution of *C. gattii* with five precipitation‐associated data layers contributing to our environmental model by more than 58% compared to only 25% for *C. neoformans*. Together, these niche models show that *C. neoformans* is associated with lower altitude and drier regions of Zambia (which is where *C. mopane* predominates) whereas *C. gattii* has a predilection for higher altitude and wetter environs (which where the Miombo *Brachystegia*‐dominated woodlands occur). More generally, for other pathogens, altitude is known to be a strong predictor of their ecotope (Messenger *et al*. 2015). Alongside altitude being a strong predictor of factors such as rainfall, altitude can also act as a physical barrier which limits gene flow and the dispersal of populations, leading to genetic isolation and speciation (Losos & Glor 2003). Investigating the factors associated with speciation may have practical implications on the potential for *Cryptococcus* species to adapt to future environments, for example, by estimating these pathogens response to climate change (Thomas *et al*. 2012).

In their natural environment, species of *Cryptococcus* do not exist in isolation and are embedded within a rich community of fungi, which are one of the most diverse groups of organisms on earth (Tedersoo *et al*. 2014). Understanding fungal community structure in the context of the distribution of *Cryptococcus* is important. This is because competitive interactions will, alongside the abiotic factors that we have here described, underlie the ability of *Cryptococcus* to proliferate as well as to invade and establish within ecoregions. Until recently, however, the sheer diversity of fungal species on earth has overwhelmed our ability to characterize their richness in nature. Here we show, using high‐throughput fungal barcoding, that ecological niche not only impacts on the distribution of the pathogenic species of *Cryptococcus* that we studied but also more broadly across the fungal kingdom in Zambia. We found that fungal community structure differed substantially across ecoregions and environmental conditions were found to be highly predictive of this structure, both seasonally and spatially (Fig. 1). Spatial and climatic factors are known to delineate organisms’ fundamental niche and therefore affect their distribution (Kozak *et al*. 2008). In support of this β−diversity (dissimilarity) analyses revealed that a fundamentally different fungal community composition occurred between the Mopane‐ and Miombo‐dominated ecoregions.

Due to the increasing movement of fungi globally, the risk of outbreaks from environmental pathogens is increasing; therefore, understanding the biotic and abiotic factors associated with such pathogens will help to foresee potential changes to their distribution and to potentially predict the emergence of disease (Wolfe *et al*. 2007; Fisher *et al*. 2012). Understanding the ecological adaptations that underpin fungal distributions will help towards a better understanding of biological mechanisms governing disease emergence (Giraud *et al*. 2010). The association of *C. gattii, C. neoformans* and the lineages therein, with different biotic and abiotic factors in Zambia underscores the adaptation of these species to different environments. Our study represents the first attempt to understand the broader community structure that is associated with a fungal pathogen species complex. We argue that understanding the ecological structuring and life‐history attributes of these pathogens are required to understand their potential to adapt to new environments, either climatic or host. Predicting disease distributions is likely to become more accurate in the near future as microbial assemblages from novel environments are becoming increasingly characterized in Big Data projects, such as the Earth Microbiome Project (http://www.earthmicrobiome.org/). Integrating macroecological analyses with population genomic data in order to link ecosystem‐level patterns to local‐scale epidemiological predictions is the ultimate goal of studies such as that which we have described here.

M.V. wrote the manuscript and performed the analyses with the help of J.R. and M.A.B. using the laboratory facilities of M.C.F., T.B. and T.S.H. implemented the clinical trial with the help of S.M., N.L., N.K., D.C., S.L. and G.K. who collected the clinical *Cryptococcus neoformans* isolates.

## Data accessibility

All raw reads and lineages information regarding each isolates have been submitted to the European Nucleotide Archive (MiSeq ITS project: PRJEB13820; WGS *Cn* Project: PRJEB13814). MiSeq ITS sample names can be found on Table S2 and isolates for WGS are listed on Table S9 (Supporting information). We welcome inquiries from all parties regarding access to cryptococcal strains or fungal sequences.

## Supporting information


**Appendix S1** Results.
**Table S1** Relative contribution of Bioclim layers to the maxent model.
**Table S2** List of sample used in ITS2 metabarcoding – The two ecoregions investigated were the Zambezi Mopane Woodlands (ZM) and the Miombo Woodlands (MW).
**Table S3** ANOSIM and permutation manova of microbial diversity patterns across Zambian ecoregions.
**Table S4** Microbial patterns within Ecoregions across seasons.
**Table S5** Permutational manova of environmental effects on microbial diversity patterns between regions.
**Table S6** BEST analysis using all ecoregion.
**Table S7** BEST analysis in Zambezi Mopane Woodlands.
**Table S8** BEST analysis Central Miombo Woodlands.
**Table S9** Environmental and clinical isolates collected in Zambia.
**Table S10** Shared SNPs between lineages and group.
**Table S11** Uniquely shared SNPs between lineages and group – single‐nucleotide polymorphism which could only be found the two groups compared.
**Table S12** *Cryptococcus gattii* (*n* = 38) recovery in Zambia.
**Table S13** Genetic diversity among the different *Cryptococcus* groups.
**Fig. S1** ITS primers with Illumina adapter overhang sequences and linker sequence.
**Fig. S2** Sampling location for each for 1391 samples collected throughout Zambia.
**Fig. S3** Fungal α‐diversity community composition of the different ecoregions – (a) Venn diagram showing the diversity of OTUs in the three ecoregions (b) rarefaction curves based on Chaos1 α‐diversity metric.
**Fig. S4** OTU distribution for MW, Miombo Woodlands, distribution of each phylum per sample.
**Fig. S5** OTU distribution for ZW, Zambian Mopane Woodlands, distribution of each phylum per sample.
**Fig. S6** OTU distribution for ZW, Zambezi Mopane Woodlands, (a) and Miombo Woodlands (MW) (b).
**Fig. S7** LDA effect size taxonomic cladogram comparing fungal community categorized by ecoregion and season.
**Fig. S8** LDA effect size ranking taxa according to effect size (highest median) and associated with season and ecoregions.
**Fig. S9** Environmental niche modelling analyses.
