# Supplementary material for: Genomic epidemiology of Cryptococcus yeasts identifies adaptation to environmental niches underpinning infection across an African HIV/AIDS cohort
Source: Mol Ecol. 2016 Nov 8;26(7):1991–2005. doi: 10.1111/mec.13891 (PMC5412878; doi:10.1111/mec.13891)

Supplementary Information

# Results

## Recovery of *Cryptococcus* in Zambia

### Zambezian Mopane Woodlands (ZM)

In the rainy season, two locations were investigated; in the Kafue strip in the Central Province (n=23, 1 positive) and near the Botswana border (n=106, 5 positives). All GIS points were recorded near *C. mopane* (n=65) and soil samples were collected near each tree (n=64). In the Kafue area, one isolate was recovered from soil and was identified as *C. gattii*. Near the Southern border, mostly Mopane trees were sampled (n=43, 3 *Cng* positives), along with *Acacia sp*. (n= *Cng* 2), *Bohinia sp*. (n=1 *Cng*), *Baobab sp.* (n=2 *Cng*), and unknown tree species (n=3 *Cng*). Two of the 56 soil samples were positive. All isolates were *C. neoformans*. Samples were also collected in Lusaka city centre (n=15), and around the town of Choma (n= 99, 5 positives). In Lusaka, all samples were taken from *Eucalyptus* trees. Around Choma, samples were collected from *Brachystegia sp.* (n=34), *C. mopane* (n=31, 4 positives), *Eucalyptus sp.* (n=3), *Acacia sp.* (n=1) *Bohinia sp.* (n=1) and unknown species (n=29, 1 positive). Three sites were found positive for *C. neoformans* (*C. mopane n=* 3) and four were for *C. gattii*. (*C. mopane* n=1; soil =3). Interestingly, one Mopane tree appeared to be positive for both *C. neoformans* and *C. gattii.* In the dry season, 12 *C. neoformans* were isolated. A total of 226 trees were sampled and 33 *C. mopane* located near the Botswana border were resampled. Two isolates were identified: one was found on a newly investigated tree and one was recovered from surrounding soils. Most of the sample were acquired by sampling the North Luangwa National Park which is dominated by *C. mopane*. Ten *C. neoformans* were recovered from trees: *C. Mopane* (n=174, 10 positives), *Eucalyptus sp.* (n=17), *Acacia sp.* (n=3), *Bohinia sp.* (n=1), unknown species (n=31). Additionally, two soil samples were positive for *C. neoformans* (n=138, 2 positive). No *C. gattii* was found. Forty-one samples were collected from Lusaka city centre. Multiple sources were sampled including trees and soil but only one *C. neoformans* isolate was isolated from soil. Additionally, 12 pigeon samples were obtained but none were positive for *C. neoformans*. The Choma region was sampled a second time (*C. mopane* n= 15; unknown species n = 27; soil n= 126), but only one *C. mopane* was positive. Molecular identification found the isolate to be *C. gattii*.

### Central Miombo Woodlands (MW)

Of the 314 miombo samples sampled in the rainy season, 17 (5.41%) were positive for *Cryptococcus gattii* and no *C. neoformans* could be found. Sampling was performed in the Copperbelt Province, principally around Kitwe and Ndola, from various sources: *Brachystegia sp.*(n=71, 2 positive), *Julbernardia globiflora* (n=9, 1 positive), *Isoberlinia angolensis* (n=6), *Eucalyptus* *sp.* (n=11), *Acacia* *sp.* (n=5), *Ficus* *sp.* (n=8), Guava tree (n=3), bamboo tree (n=3), mango tree (n=1), and unknown tree species (n=42, 2 positive). Five samples were recovered from animals: chicken (n=2), Red Necked Francolin (n=3). Five isolates were recovered from soil. Cracks in granite kopjes were also investigated. Most of them showed the presence of animal droppings from rock hyrax. Of these cracks (n=34), seven were positive for *Cryptococcus gattii* (20.6%). In the dry season, 100 samples were obtained in the MW, yielding two *C. neoformans* and 15 *C. gattii*. The latter was identified in various tree species including: *Brachystegia sp.* (n=52, 4 positive), *Julbernardia globiflora* (n=15, 1 positive), *Isoberlinia angolensis* (n=3), *Eucalyptus* (n=12, 2 positive). Additionally, isolates were found in soil (n=45, 4 positives) and Hyrax middens (n=18, 4 positives). Two *C. neoformans* were also recovered from soil.

## Association between Environmental Factors and Fungal Community Structure

To determine the role of environmental factors on the β-diversity of fungal communities, permutational MANOVA and a best variables rank correlation test (BEST) analysis was performed. The environmental factors investigated were the Bioclim layers (Busby, 1991) available on the WorldClim website (<http://www.worldclim.org/bioclim>). Between ecoregions, all environmental metrics displayed significant degrees of correlation with respect to fungal diversity, except for soil properties. The MANOVA tests identified ‘Precipitation of Wettest Quarter’ (bio16) (R^2^=2.6363, *p*<0.001) to be most significant variable to influence fungal communities (Table S5). Then, followed ‘Precipitation of Warmest Quarter’ (bio18) (R^2^=1.5652, *p*<0.001), ‘Precipitation of Driest Month’ (Bio14) (R^2^ = 1.4642, *p* < 0.001), ‘Temperature Annual Range’ (Bio7) (R^2^=1.4642, *p*<0.001) and ‘Mean Diurnal Range’ (Bio2) (R^2^= 1.4629, *p* < 0.001). Climatic variables appear to have a more significant influence on β-diversity than geographical variables. In support of this, the MANOVA R^2^ coefficient of elevation, latitude and longitude was the lowest (R^2^ < 1.12, *p* < 0.001) but all features appeared significant. BEST analysis determines which environmental feature explains the greatest degree of fungal diversity. This method ranked ‘Precipitation of Wettest Month’ (bio13) as the highest correlation between the diversity distance-matrix. Then ‘Mean Temperature of Warmest Quarter’ (bio10) and elevation were the second and third most significant factors (Table S6).

Within the ZM, only ‘Min Temperature of Coldest Month’ (bio6) (R^2^ = 1.1109, *p* < 0.01) and ‘Mean Temperature of Wettest Quarter’ (bio8) (R^2^ = 1.1659, *p* = 0.042) were significant with MANOVA tests. The BEST analyses identified ‘Temperature of Wettest Quarter’ (bio8) as most important feature confirming the MANOVA tests. ‘Isothermality’ (Bio3) and ‘Mean Temperature of Warmest Quarter’ (bio10) were also found to be important contributing factors (Table S7). In the MW, ‘Precipitation Seasonality’ (bio15) (R^2^ = 1.4653, *p* = 0.003) was the most significant variable in the MANOVA tests. Other features such as ‘Annual Mean Temperature’ (bio1), ‘Precipitation of Wettest Quarter’ (bio16), ‘Temperature Seasonality’ (bio4) and ‘Precipitation of Wettest Month’ (bio13) were also significant. BEST analysis confirmed the role of ‘Precipitation Seasonality’ (bio15) as the main feature to explain differences in microbial diversity but the analysis emphasised the role of elevation and latitude on fungal β-diversity (Table S8). BEST analysis agrees our environmental niche model and confirms the role of temperature and atmospheric conditions, mainly precipitations, on the distribution on fungal species.

## Supplementary Tables:

| Climatic Variables | | Relative Contribution (%) | |
| --- | --- | --- | --- |
| Number | Name | *Cng* | *Cg* |
| bio1 | Annual Mean Temperature | 0 | 0 |
| bio2 | Mean Diurnal Range | 0 | 0 |
| bio3 | Isothermality | 71.2 | 26.5 |
| bio4 | Temperature Seasonality | 0 | 0 |
| bio5 | Max Temperature of Warmest Month | 0.4 | 0 |
| bio6 | Min Temperature of Coldest Month | 0 | 3 |
| bio7 | Temperature Annual Range | 3.3 | 0 |
| bio8 | Mean Temperature of Wettest Quarter | 0 | 0.2 |
| bio9 | Mean Temperature of Driest Quarter | 0 | 7.9 |
| bio10 | Mean Temperature of Warmest Quarter | 0 | 0 |
| bio11 | Mean Temperature of Coldest Quarter | 0 | 5.1 |
| bio12 | Annual Precipitation | 0 | 0 |
| bio13 | Precipitation of Wettest Month | 0 | 3.9 |
| bio14 | Precipitation of Driest Month | 2.6 | 9.1 |
| bio15 | Precipitation Seasonality | 22 | 4.5 |
| bio16 | Precipitation of Wettest Quarter | 0 | 18.4 |
| bio17 | Precipitation of Driest Quarter | 0.5 | 5.6 |
| bio18 | Precipitation of Warmest Quarter | 0 | 16.8 |
| bio19 | Precipitation of Coldest Quarter | 0 | 0 |
| Alt | Altitude | 0 | 3.2 |

Table S1 - Relative contribution of Bioclim layers to the MaxEnt model

| SampleID | Date | Season | Ecoregions | Latitude | Longitude | Elevation | OTUs | Cng/Cg |
| --- | --- | --- | --- | --- | --- | --- | --- | --- |
| BM06 | 19/01/2013 | Rainy | ZM | -16.65 | 27.042 | 1221 | 227 | - |
| BM07w | 19/01/2013 | Rainy | ZM | -16.65 | 27.043 | 1216 | 81 | - |
| BM17 | 19/01/2013 | Rainy | ZM | -16.65 | 27.043 | 1216 | 176 | Cg |
| BM7d | 19/01/2013 | Rainy | ZM | -16.65 | 27.043 | 1216 | 245 | - |
| BMS03w | 19/01/2013 | Rainy | ZM | -16.651 | 27.042 | 1224 | 239 | - |
| BMS08 | 19/01/2013 | Rainy | ZM | -16.65 | 27.043 | 1216 | 235 | *Cng/Cg* |
| BMS11 | 19/01/2013 | Rainy | ZM | -16.649 | 27.044 | 1215 | 113 | - |
| BMS31w | 20/01/2013 | Rainy | ZM | -16.624 | 26.974 | 1213 | 128 | - |
| BMS47 | 20/01/2013 | Rainy | ZM | -16.626 | 26.977 | 1218 | 97 | - |
| BMS53 | 21/01/2013 | Rainy | ZM | -16.642 | 27.027 | 1188 | 128 | - |
| EucY9 | 21/01/2013 | Rainy | ZM | -16.642 | 27.027 | 1188 | 153 | *Cng* |
| G16 | 14/01/2013 | Rainy | MW | -12.934 | 28.26 | 1189 | 256 | - |
| G18 | 14/01/2013 | Rainy | MW | -12.933 | 28.259 | 1188 | 278 | *Cg* |
| GR33 | 15/01/2013 | Rainy | MW | -12.945 | 28.323 | 1216 | 173 | - |
| GRS19 | 14/01/2013 | Rainy | MW | -12.933 | 28.259 | 1186 | 80 | - |
| GRS31 | 15/01/2013 | Rainy | MW | -12.945 | 28.324 | 1217 | 89 | *Cg* |
| GRS34 | 15/01/2013 | Rainy | MW | -12.944 | 28.323 | 1215 | 133 | - |
| GRS51 | 16/01/2013 | Rainy | MW | -12.994 | 28.24 | 1219 | 128 | - |
| GRS52 | 16/01/2013 | Rainy | MW | -12.994 | 28.24 | 1222 | 115 | - |
| GRS58 | 16/01/2013 | Rainy | MW | -12.995 | 28.241 | 1212 | 164 | *Cg* |
| GRS59 | 16/01/2013 | Rainy | MW | -12.994 | 28.241 | 1214 | 138 | - |
| GRS64 | 16/01/2013 | Rainy | MW | -12.995 | 28.241 | 1212 | 179 | *Cg* |
| GRS69 | 16/01/2013 | Rainy | MW | -12.993 | 28.24 | 1190 | 113 | *Cg* |
| GRS70 | 16/01/2013 | Rainy | MW | -12.993 | 28.24 | 1181 | 67 | - |
| GRS72 | 16/01/2013 | Rainy | MW | -12.993 | 28.24 | 1181 | 102 | - |
| GRS88I | 17/01/2013 | Rainy | MW | -12.926 | 28.242 | 1170 | 139 | - |
| GRS92 | 17/01/2013 | Rainy | MW | -12.927 | 28.245 | 1179 | 222 | - |
| GRS98 | 17/01/2013 | Rainy | MW | -12.927 | 28.246 | 1187 | 0 | *Cg* |
| H10 | 17/09/2013 | Dry | MW | -12.927 | 28.246 | 1187 | 82 | *Cg* |
| H2 | 17/09/2013 | Dry | MW | -12.927 | 28.245 | 1172 | 340 |  |
| H23 | 17/09/2013 | Dry | MW | -12.927 | 28.245 | 1172 | 0 | - |
| H26 | 17/09/2013 | Dry | MW | -12.927 | 28.245 | 1172 | 182 | - |
| H44 | 17/09/2013 | Dry | MW | -12.927 | 28.245 | 1172 | 297 | - |
| H7 | 17/09/2013 | Dry | MW | -12.927 | 28.245 | 1172 | 327 | *Cg* |
| I13 | 17/09/2013 | Dry | MW | -12.927 | 28.245 | 1172 | 182 | - |
| I2 | 17/09/2013 | Dry | MW | -12.927 | 28.245 | 1172 | 43 | *Cg* |
| I3 | 17/09/2013 | Dry | MW | -12.927 | 28.245 | 1172 | 289 | - |
| K14 | 23/01/2013 | Rainy | MW | -15.844 | 28.239 | 984 | 144 | *Cg* |
| K2 | 23/01/2013 | Rainy | MW | -15.844 | 28.239 | 984 | 177 | *Cg* |
| K7 | 23/01/2013 | Rainy | MW | -15.843 | 28.24 | 983 | 189 | - |
| NL13 | 23/01/2013 | Rainy | MW | -15.843 | 28.239 | 986 | 179 | *Cng* |
| NL21 | 22/09/2013 | Dry | ZM | -11.753 | 32.306 | 702 | 382 | - |
| NL24 | 22/09/2013 | Dry | ZM | -11.753 | 32.306 | 702 | 320 | - |
| NL36 | 22/09/2013 | Dry | ZM | -11.753 | 32.306 | 702 | 182 | - |
| NL38 | 22/09/2013 | Dry | ZM | -11.753 | 32.306 | 702 | 197 | *Cng* |
| NL57 | 22/09/2013 | Dry | ZM | -11.753 | 32.306 | 702 | 187 | - |
| NL65 | 22/09/2013 | Dry | ZM | -11.753 | 32.306 | 702 | 281 | - |
| NL9 | 22/09/2013 | Dry | ZM | -11.753 | 32.306 | 702 | 248 | - |
| O23 | 22/09/2013 | Dry | ZM | -11.753 | 32.306 | 702 | 249 | *Cng* |
| O43 | 23/09/2013 | Dry | ZM | -11.868 | 32.436 | 589 | 206 | - |
| O57 | 23/09/2013 | Dry | ZM | -11.868 | 32.436 | 589 | 283 | - |
| O8 | 23/09/2013 | Dry | ZM | -11.868 | 32.436 | 904 | 213 | - |
| V16 | 23/09/2013 | Dry | ZM | -17.842 | 25.786 | 589 | 155 | - |
| V18 | 22/01/2013 | Rainy | ZM | -17.842 | 25.786 | 904 | 165 | *Cng* |
| V18d | 22/01/2013 | Rainy | ZM | -17.842 | 25.786 | 904 | 165 | - |
| V31bis | 22/01/2013 | Rainy | ZM | -17.842 | 25.786 | 904 | 159 | *Cng* |
| VF3 | 23/01/2013 | Rainy | ZM | -17.842 | 25.786 | 904 | 216 | - |
| VF5A | 23/01/2013 | Rainy | ZM | -17.842 | 25.786 | 904 | 13 | - |
| VF9A | 23/01/2013 | Rainy | ZM | -17.842 | 25.786 | 904 | 69 | - |
| VS17 | 21/01/2013 | Rainy | ZM | -17.898 | 25.85 | 890 | 171 | - |
| VS2 | 21/01/2013 | Rainy | ZM | -17.899 | 25.856 | 896 | 489 | - |
| VS21 | 21/01/2013 | Rainy | ZM | -17.899 | 25.856 | 896 | 325 | - |
| VS21bis | 21/01/2013 | Rainy | ZM | -17.899 | 25.856 | 896 | 0 | - |
| VS24 | 21/01/2013 | Rainy | ZM | -17.899 | 25.856 | 896 | 132 | *Cng/Cg* |
| VS31 | 21/01/2013 | Rainy | ZM | -17.899 | 25.856 | 896 | 216 | - |
| VS37A | 21/01/2013 | Rainy | ZM | -17.899 | 25.856 | 896 | 132 | - |
| VS5 | 21/01/2013 | Rainy | ZM | -17.899 | 25.856 | 896 | 242 | - |
| W13 | 28/09/2013 | Dry | ZM | -17.842 | 25.786 | 904 | 113 | - |
| W14 | 28/09/2013 | Dry | ZM | -17.842 | 25.786 | 904 | 192 | - |
| W20 | 28/09/2013 | Dry | ZM | -17.842 | 25.786 | 904 | 362 | - |
| W22 | 28/09/2013 | Dry | ZM | -17.842 | 25.786 | 904 | 163 | - |
| W29 | 28/09/2013 | Dry | ZM | -17.842 | 25.786 | 904 | 270 | - |
| W44 | 28/09/2013 | Dry | ZM | -17.842 | 25.786 | 904 | 239 | - |

Table S2: List of sample used in ITS2 metabarcoding – The two ecoregions investigated were the Zambezi Mopane Woodlands (ZM) and the Miombo Woodlands (MW).

|  |  | Bray-curtis | | | |
| --- | --- | --- | --- | --- | --- |
|  |  | ANOSIM | | ADONIS | |
| Group | Factor | R | *p* | R^2^ | *p* |
| Ecoregions | Ecoregions | 0.3704 | 0.001 | 0.0687 | 0.001 |
| Ecoregions | Elevation | 0.4272 | 0.001 | 0.0374 | 0.001 |
| Ecoregions | Longitude | 0.4191 | 0.001 | 0.0183 | 0.198 |
| Ecoregions | Latitude | 0.4184 | 0.001 | 0.0242 | 0.006 |
| Ecoregions | Season | 0.3744 | 0.001 | 0.0687 | 0.001 |

Table S3 – ANOSIM and permutation MANOVA of microbial diversity patterns across Zambian Ecoregions

|  | Zambezi Mopane Woodlands (ZM) | | | | Miombo Woodlands (MW) | | | |
| --- | --- | --- | --- | --- | --- | --- | --- | --- |
| Season | Rainy | | Dry | | Rainy | | Dry | |
| Phylum | Seq. | % | Seq. | % | Seq. | % | Seq. | % |
| Ascomycota | 70.22 | 26.36% | 67.95 | 33.89% | 58.56 | 29.41% | 167.38 | 50.30% |
| Basidiomycota | 49.89 | 10.50% | 10.26 | 9.98% | 28.17 | 8.59% | 95.88 | 13.44% |
| Chytridiomycota | 9 | 5.96% | 10.21 | 3.04% | 4.39 | 2.15% | 18.13 | 4.36% |
| Glomeromycota | 7.89 | 0.33% | 5.95 | 0.28% | 3.94 | 2.88% | 14.25 | 0.51% |
| Zygomycota | 5.44 | 15.86% | 19 | 27.11% | 2.5 | 7.33% | 9.13 | 8.09% |
| Other | - | 3.57% | - | 0.47% | - | 2.18 | - | 0.02% |
| Unidentified | - | 37.42% | - | 25.22% | - | 47.47% | - | 23.27% |
| Total | 145.33 | 100.00% | 143.37 | 100.00% | 137.67 | 100.00% | 221.63 | 100.00% |

TableS4 - Microbial patterns within Ecoregions across seasons

|  | Bray-curtis | | | | | |
| --- | --- | --- | --- | --- | --- | --- |
|  | Ecoregions | | Mopane Woodlands | | Miombo Woodlands | |
|  | R^2^ | *p* | R^2^ | *p* | R^2^ | *p* |
| Longitude | 1.1185 | 0.001 | 1.0811 | 0.114 | 1.032 | 0.209 |
| Latitude | 1.1185 | 0.003 | 1.0811 | 0.112 | 1.032 | 0.187 |
| Elevation | 1.1171 | 0.002 | 1.0734 | 0.14 | 1.032 | 0.186 |
| EVI | 1.1871 | 0.01 | 1.0811 | 0.11 | 1.0552 | 0.18 |
| NDVI | 1.165 | 0.01 | 0.144 | 0.08 | NA | NA |
| Bio1 | 1.3712 | 0.001 | 1.1109 | 0.069 | 1.1455 | 0.048 |
| Bio2 | 1.4629 | 0.003 | 1.1109 | 0.093 | 1.1119 | 0.116 |
| Bio3 | 1.2988 | 0.001 | 1.174 | 0.128 | NA | NA |
| Bio4 | 1.3443 | 0.001 | 1.1109 | 0.084 | 1.1455 | 0.046 |
| Bio5 | 1.3996 | 0.001 | 1.1109 | 0.088 | 0.9954 | 0.484 |
| Bio6 | 1.4009 | 0.001 | 1.1109 | 0.01 | 1.2599 | 0.008 |
| Bio7 | 1.4642 | 0.001 | 1.1109 | 0.068 | 1.2338 | 0.016 |
| Bio8 | 1.3217 | 0.001 | 1.1659 | 0.042 | 0.9954 | 0.434 |
| Bio9 | 1.3147 | 0.001 | 1.1109 | 0.086 | 0.9954 | 0.477 |
| Bio10 | 1.3217 | 0.001 | 1.1323 | 0.061 | 0.9954 | 0.489 |
| Bio11 | 1.2988 | 0.001 | 1.1109 | 0.079 | 0.9954 | 0.476 |
| Bio12 | 1.3704 | 0.001 | 1.1109 | 0.083 | 1.1455 | 0.057 |
| Bio13 | NA | NA | 1.1323 | 0.068 | 1.1455 | 0.05 |
| Bio14 | 1.4642 | 0.001 | 1.1323 | 0.066 | NA | NA |
| Bio15 | 1.3417 | 0.001 | 1.1323 | 0.062 | 1.4653 | 0.003 |
| Bio16 | 2.6363 | 0.001 | 1.1109 | 0.098 | 1.1455 | 0.037 |
| Bio17 | 1.3579 | 0.001 | 1.2019 | 0.08 | NA | NA |
| Bio18 | 1.5652 | 0.001 | 1.1109 | 0.089 | 1.1455 | 0.066 |
| Bio19 | NA | NA | 1.2019 | 0.081 | 1.0421 | 0.339 |

Table S5 - Permutational MANOVA of environmental effects on microbial diversity patterns between regions

| **Bray-Curtis Dissimilarity** | |  |
| --- | --- | --- |
| size | *p* | Variables |
| 1 | 0.303 | bio13 |
| 2 | 0.335 | bio10, bio13 |
| 3 | 0.338 | Elevation, bio12, bio13 |
| 4 | 0.335 | bio1, bio8, bio12, bio13 |
| 5 | 0.331 | bio1, bio8, bio10, bio12, bio13 |
| 6 | 0.327 | Elevation, bio5, bio8, bio10, bio12, bio13 |
| 7 | 0.324 | Elevation, bio1, bio5, bio8, bio10, bio12, bio13 |
| 8 | 0.292 | Elevation, bio1, bio5, bio8, bio10, bio11, bio12, bio13 |
| 9 | 0.281 | Elevation, bio1, bio5, bio8, bio9, bio10, bio11, bio12, bio13 |
| 10 | 0.266 | Elevation, Latitude, bio1, bio3, bio5, bio8, bio10, bio11, bio12, bio13 |
| 11 | 0.256 | Elevation, Latitude, bio1, bio3, bio4, bio5, bio8, bio10, bio11, bio12, bio13 |
| 12 | 0.240 | Elevation, Latitude, Longitude, bio1, bio3, bio5, bio6, bio8, bio9, bio10, bio11, bio13 |
| 13 | 0.236 | Elevation, Latitude, bio1, bio2, bio3, bio5, bio6, bio8, bio9, bio10, bio11, bio12, bio13 |
| 14 | 0.229 | Elevation, Latitude, Longitude, bio1, bio2, bio3, bio5, bio6, bio8, bio9, bio10, bio11, bio12, bio13 |
| 15 | 0.197 | Elevation, Latitude, bio1, bio2, bio3, bio4, bio5, bio6, bio7, bio8, bio9, bio10, bio11, bio12, bio13 |
| 16 | 0.122 | Elevation, Latitude, Longitude, bio1, bio2, bio3, bio4, bio5, bio6, bio7, bio8, bio9, bio10, bio11, bio12, bio13 |

Table S6 - BEST analysis using all Ecoregion

| **Bray-Curtis Dissimilarity** | |  |
| --- | --- | --- |
| size | p | Variables |
| 1 | 0.234 | bio8 |
| 2 | 0.222 | bio3, bio8 |
| 3 | 0.222 | bio3, bio8, bio10 |
| 4 | 0.218 | bio2, bio3, bio4, bio8 |
| 5 | 0.220 | bio5, bio7, bio8, bio16, bio18 |
| 6 | 0.221 | Latitude, Elevation, bio4, bio5, bio8, bio18 |
| 7 | 0.222 | Latitude, Elevation, bio2, bio4, bio5, bio8, bio18 |
| 8 | 0.222 | Latitude, Elevation, bio2, bio3, bio4, bio5, bio8, bio18 |
| 9 | 0.222 | Latitude, Elevation, bio2, bio4, bio5, bio7, bio10, bio16, bio18 |
| 10 | 0.222 | Latitude, Elevation, bio2, bio4, bio5, bio7, bio8, bio10, bio16, bio18 |
| 11 | 0.222 | Latitude, Elevation, bio2, bio4, bio5, bio6, bio7, bio8, bio10, bio16, bio18 |
| 12 | 0.221 | Latitude, Longitude, Elevation, bio2, bio3, bio4, bio5, bio6, bio8, bio10, bio16, bio18 |
| 13 | 0.221 | Latitude, Longitude, Elevation, bio2, bio3, bio4, bio5, bio6, bio7, bio8, bio10, bio16, bio18 |
| 14 | 0.217 | Latitude, Longitude, Elevation, bio2, bio3, bio4, bio5, bio6, bio7, bio8, bio9, bio10, bio16, bio18 |
| 15 | 0.205 | Latitude, Longitude, Elevation, bio2, bio3, bio4, bio5, bio6, bio7, bio8, bio9, bio10, bio15, bio16, bio18 |
| 16 | 0.195 | Latitude, Longitude, Elevation, bio1, bio2, bio3, bio4, bio5, bio6, bio7, bio8, bio9, bio10, bio15, bio16, bio18 |

Table S7 - BEST analysis in Zambezi Mopane Woodlands

| **Bray-Curtis Dissimilarity** | |  |
| --- | --- | --- |
| size | *p* | Variables |
| 1 | 0.230 | bio15 |
| 2 | 0.225 | Latitude, bio16 |
| 3 | 0.234 | Latitude, Elevation, bio15 |
| 4 | 0.234 | Latitude, Elevation, bio15, bio16 |
| 5 | 0.242 | Latitude, Elevation, bio15, bio16, bio18 |
| 6 | 0.238 | Latitude, Elevation, bio6, bio15, bio16, bio18 |
| 7 | 0.228 | Latitude, Elevation, bio1, bio6, bio15, bio16, bio18 |
| 8 | 0.205 | Latitude, Elevation, bio1, bio6, bio7, bio15, bio16, bio18 |
| 9 | 0.188 | Latitude, Elevation, bio1, bio5, bio6, bio8, bio15, bio16, bio18 |
| 10 | 0.174 | Latitude, Elevation, bio1, bio2, bio4, bio6, bio7, bio9, bio15, bio18 |
| 11 | 0.171 | Latitude, Longitude, Elevation, bio1, bio2, bio4, bio5, bio7, bio15, bio16, bio18 |
| 12 | 0.171 | Latitude, Longitude, Elevation, bio1, bio2, bio5, bio6, bio7, bio8, bio15, bio16, bio18 |
| 13 | 0.171 | Latitude, Longitude, Elevation, bio1, bio2, bio4, bio5, bio6, bio7, bio8, bio15, bio16, bio18 |
| 14 | 0.161 | Latitude, Elevation, bio1, bio2, bio4, bio5, bio6, bio7, bio8, bio9, bio10, bio15, bio16, bio18 |
| 15 | 0.144 | Latitude, Longitude, Elevation, bio1, bio2, bio4, bio5, bio6, bio7, bio8, bio9, bio10, bio15, bio16, bio18 |

### Table S8 - BEST analysis Central Miombo Woodlands

| Isolates | Lineage | Country | MAT | Origin | Environment | Latitude | Longitude | Mapped reads (%) |
| --- | --- | --- | --- | --- | --- | --- | --- | --- |
| Ze1 | VNB-A | Zambia | α | *C. mopane* | Rural | -16.65 | 27.04 | 91% |
| Ze2 | VNB-B | Zambia | α | *C. mopane* | Rural | -16.63 | 26.98 | 87% |
| Ze3 | VNB-B | Zambia | a | *C. mopane* | Rural | -17.89 | 25.86 | 84% |
| Ze4 | VNB-A | Zambia | a | *C. mopane* | Rural | -17.84 | 25.79 | 83% |
| Ze5 | VNB-A | Zambia | a | *C. mopane* | Rural | -17.85 | 25.8 | 76% |
| Ze6 | VNB-B | Zambia | α | Soil | Rural | -17.83 | 25.77 | 88% |
| Ze7 | VNB-A | Zambia | α | Soil | Rural | -17.84 | 25.79 | 86% |
| Ze8 | VNI | Zambia | α | *Eucalyptus sp.* | Urban | -15.51 | 28.27 | 92% |
| Ze9 | VNB-B | Zambia | α | *C. mopane* | Rural | -17.9 | 25.86 | 89% |
| Ze10 | VNB-B | Zambia | α | *C. mopane* | Rural | -17.83 | 25.77 | 88% |
| Ze11 | VNI | Zambia | α | *C. mopane* | Rural | -17.83 | 25.77 | 94% |
| Ze12 | VNI | Zambia | α | *C. mopane* | Rural | -17.85 | 25.79 | 94% |
| Ze13 | VNI | Zambia | α | *C. mopane* | Rural | -17.89 | 25.86 | 94% |
| Ze14 | VNB-B | Zambia | a | *Brachestegia sp.* | Rural | -12.93 | 28.25 | 90% |
| Ze15 | VNB-B | Zambia | a | *C. mopane* | Rural | -11.73 | 32.14 | 90% |
| Ze16 | VNB-B | Zambia | a | *C. mopane* | Rural | -11.75 | 32.31 | 91% |
| Ze17 | VNB-B | Zambia | α | *C. mopane* | Rural | -11.87 | 32.44 | 85% |
| Ze18 | VNB-B | Zambia | α | *C. mopane* | Rural | -11.87 | 32.44 | 86% |
| Ze19 | VNB-B | Zambia | α | *C. mopane* | Rural | -13.04 | 31.92 | 90% |
| Ze20 | VNB-A | Zambia | a | *Brachestegia sp.* | Rural | -14.25 | 31.68 | 75% |
| Ze21 | VNB-B | Zambia | α | *C. mopane* | Rural | -11.92 | 32.26 | 82% |
| Ze22 | VNB-B | Zambia | α | *C. mopane* | Rural | -11.92 | 32.26 | 88% |
| Ze23 | VNB-B | Zambia | α | *C. mopane* | Rural | -11.92 | 32.26 | 87% |
| Ze24 | VNI | Zambia | α | Soil | Rural | -13.12 | 28.18 | 93% |
| Zc01 | VNI | Zambia | α | Patient | Clinical | -15.41 | 28.31 | 97% |
| Zc02 | VNI | Lusaka | α | Patient | Clinical | -15.41 | 28.31 | 97% |
| Zc04 | VNB-A | Lusaka | α | Patient | Clinical | -15.41 | 28.31 | 94% |
| Zc05 | VNI | Lusaka | α | Patient | Clinical | -15.41 | 28.31 | 94% |
| Zc06 | VNII | Lusaka | α | Patient | Clinical | -15.41 | 28.31 | 92% |
| Zc07 | VNB-A | Lusaka | α | Patient | Clinical | -15.41 | 28.31 | 94% |
| Zc10 | VNI | Lusaka | α | Patient | Clinical | -15.41 | 28.31 | 97% |
| Zc12 | VNI | Lusaka | α | Patient | Clinical | -15.41 | 28.31 | 97% |
| Zc13 | VNI | Lusaka | α | Patient | Clinical | -15.41 | 28.31 | 98% |
| Zc14 | VNII | Lusaka | α | Patient | Clinical | -15.41 | 28.31 | 93% |
| Zc15 | VNB-A | Lusaka | α | Patient | Clinical | -15.41 | 28.31 | 92% |
| Zc16 | VNI | Lusaka, alpha | α | Patient | Clinical | -15.41 | 28.31 | 96% |
| Zc18 | VNI | Lusaka | α | Patient | Clinical | -15.41 | 28.31 | 94% |
| Zc21 | VNB-A | Copperbelt | α | Patient | Clinical | -13.11 | 27.84 | 93% |
| Zc23 | VNI | Lusaka | α | Patient | Clinical | -15.41 | 28.31 | 94% |
| Zc24 | VNI | Kitwe | α | Patient | Clinical | -12.82 | 28.21 | 94% |
| Zc26 | VNI | Lusaka | α | Patient | Clinical | -15.41 | 28.31 | 98% |
| Zc27 | VNI | Kafue | α | Patient | Clinical | -15.76 | 28.18 | 97% |
| Zc28 | VNI | Kafue | α | Patient | Clinical | -15.76 | 28.18 | 96% |
| Zc29 | VNI | Lusaka | α | Patient | Clinical | -15.41 | 28.31 | 96% |
| Zc30 | VNI | Kitwe | α | Patient | Clinical | -12.82 | 28.21 | 98% |
| Zc32 | VNII | Copperbelt, Lusaka | α | Patient | Clinical | -13.11 | 27.84 | 93% |
| Zc34 | VNI | Copperbelt | α | Patient | Clinical | -13.11 | 27.84 | 96% |

Table S9 – Environmental and clinical isolates collected in Zambia. The average read mapped to the reference genome for the VNI molecular type was 95%, while 87% of reads could be mapped to the VNI reference genome among VNB isolates.

| Shared SNPs | VNI Clinical | VNI Environmental | VNB Clinical | VNB Environmental | VNII Clinical |
| --- | --- | --- | --- | --- | --- |
| VNI Clinical (n=16) | - |  |  |  |  |
| VNI Environmental (n=5) | 75,793 | - |  |  |  |
| VNB Clinical (n=4) | 42,385 | 34,096 | - |  |  |
| VNB Environmental (n=19) | 47,727 | 38,097 | 292,515 | - |  |
| VNII (n=3) | 40,193 | 33,021 | 187,049 | 203,452 | - |

Table S10 –Shared SNPs between lineages and group

| Uniquely shared SNPs | VNI Clinical | VNI Environmental | VNB Clinical | VNB Environmental | VNII Clinical |
| --- | --- | --- | --- | --- | --- |
| VNI Clinical (n=16) | - |  |  |  |  |
| VNI Environmental (n=5) | 34,996 | - |  |  |  |
| VNB Clinical (n=4) | 289 | 9 | - |  |  |
| VNB Environmental (n=19) | 1,360 | 111 | 104,902 | - |  |
| VNII (n=3) | 558 | 35 | 4,900 | 19,992 | - |

Table S11 – Uniquely shared SNPs between lineages and group – single-nucleotide polymorphism which could only be found the two groups compared.

| Name | **Species** | **Source** | **Tree Species** | **Date** | **Ecoregions** | **Latitude** | **Longitude** |
| --- | --- | --- | --- | --- | --- | --- | --- |
| BM17-1 | *Cg* | Tree | *Unknown* | 19/01/2013 | ZM | -16.65 | 27.04 |
| P17-2 | *Cg* | Tree | *C. mopane* | 25/09/2013 | ZM | -13.04 | 31.92 |
| GR31-1 | *Cg* | Tree | Fig Tree | 15/01/2013 | MW | -12.94 | 28.32 |
| GR53-1 | *Cg* | Rock cove | *Hyrax midden* | 16/01/2013 | MW | -12.99 | 28.24 |
| GR57-4 | *Cg* | Rock cove | *Hyrax midden* | 16/01/2013 | MW | -12.99 | 28.24 |
| GR65-5 | *Cg* | Tree | *Julbernadia globiflora* | 16/01/2013 | MW | -12.99 | 28.24 |
| GR67-5 | *Cg* | Rock cove | *Hyrax midden* | 16/01/2013 | MW | -12.99 | 28.24 |
| GR67D-7 | *Cg* | Rock cove | *Hyrax midden* | 16/01/2013 | MW | -12.99 | 28.24 |
| GR70D-2 | *Cg* | Rock cove | *Hyrax midden* | 16/01/2013 | MW | -12.99 | 28.24 |
| GR79D-3 | *Cg* | Tree | *Eucalyptus* | 17/01/2013 | MW | -12.93 | 28.24 |
| GRS53 | *Cg* | Soil | - | 16/01/2013 | MW | -12.99 | 28.24 |
| GRS54-2 | *Cg* | Soil | - | 16/01/2013 | MW | -12.99 | 28.24 |
| GRS58-2 | *Cg* | Soil | *Eucalyptus* | 16/01/2013 | MW | -12.99 | 28.24 |
| GRS61 | *Cg* | Rock cove | *Hyrax midden* | 16/01/2013 | MW | -12.99 | 28.24 |
| GRS64-4 | *Cg* | Rock cove | *Hyrax midden* | 16/01/2013 | MW | -12.99 | 28.24 |
| GR67D-3 | *Cg* | Rock cove | *Hyrax midden* | 16/01/2013 | MW | -12.99 | 28.24 |
| GRS70-1 | *Cg* | Rock cove | *Hyrax midden* | 16/01/2013 | MW | -12.99 | 28.24 |
| GR98-1 | *Cg* | Soil | - | 17/01/2013 | MW | -12.93 | 28.25 |
| BM08A1 | *Cg* | Tree | *C. mopane* | 19/01/2013 | ZM | -16.65 | 27.04 |
| BMS08A-1 | *Cg* | Soil | - | 19/01/2013 | ZM | -16.65 | 27.04 |
| BM09-13 | *Cg* | Soil | - | 19/01/2013 | ZM | -16.65 | 27.04 |
| Z5-10 | *Cg* | Tree | *Unknown* | 25/11/2012 | MW |  |  |
| VS24A1-bis | *Cg* | Tree | *C. mopane* | 02/09/2013 | ZM | -17.83 | 25.77 |
| BM*08B1 | *Cg* | Soil | - | 08/09/2013 | MW | -16.65 | 27.04 |
| H5-1 | *Cg* | Rock cove | *Hyrax midden* | 17/09/2013 | MW | -13.12 | 28.19 |
| H7-2 | *Cg* | Rock cove | *Hyrax midden* | 17/09/2013 | MW | -13.12 | 28.19 |
| H10-2 | *Cg* | Rock cove | *Hyrax midden* | 17/09/2013 | MW | -13.12 | 28.19 |
| H11-1 | *Cg* | Rock cove | *Hyrax midden* | 17/09/2013 | MW | -13.12 | 28.19 |
| H44-4 | *Cg* | Tree | *Brachestesia* | 17/09/2013 | MW | -12.93 | 28.25 |
| K1-1 | *Cg* | Tree | *Brachestesia* | 20/09/2013 | MW | -12.45 | 31.29 |
| K2-1 | *Cg* | Tree | *Brachestesia* | 20/09/2013 | MW | -12.45 | 31.29 |
| K13-2 | *Cg* | Tree | *Hyrax midden* | 20/09/2013 | MW | -11.19 | 31.59 |
| K14-6 | *Cg* | Tree | *Brachestesia* | 20/09/2013 | MW | -11.17 | 31.61 |
| KS14-1 | *Cg* | Soil | - | 20/09/2013 | MW | -11.17 | 31.61 |
| P17-1 | *Cg* | Tree | *C. mopane* | 25/09/2013 | MW | -13.04 | 31.92 |
| I2-4 | *Cg* | Tree bark | *Brachestesia* | 18/09/2013 | MW | -13.63 | 28.62 |
| G18-1 | *Cg* | Tree bark | *Brachestesia* | 18/09/2013 | MW | -12.48 | 31.32 |
| HS10-3 | *Cg* | Rock cove | *Hyrax midden* | 17/09/2013 | MW | -13.12 | 28.19 |

Table S12 – *Cryptococcus gattii* (n=38) recovery in Zambia

|  | S | η | ηE | π | θ | Tajima’s *D* |
| --- | --- | --- | --- | --- | --- | --- |
| VNB (n=23) | 521,924 | 527,744 | 197,262 | 0.219 | 0.234 | -0.265 |
| VNB Environmental (n=19) | 440,457 | 444,573 | 160,841 | 0.229 | 0.238 | -0.161 |
| VNB Clinical (n=3) | 233,414 | 234,347 | 176,424 | 0.344 | 0.347 | -0.095 |
| VNI (n=21) | 105,391 | 105,586 | 16,899 | 0.337 | 0.278 | 0.884 |
| VNI Environmental (n=4) | 51,762 | 51,799 | 37,216 | 0.306 | 0.322 | -0.379 |
| VNI Clinical (n=15) | 103,996 | 104,181 | 22,903 | 0.347 | 0.302 | 0.663 |
| VNII (n=3) | 366,086 | 367,794 | 283,200 | 0.540 | 0.548 | -0.152 |

Table S13: Genetic diversity among the different *Cryptococcus* groups. S = segregating sides; η = number of mutations; ηE = number of external mutations; π = nucleotide differences per site; θ = Watterson’s estimate of the population scaled mutation rate.


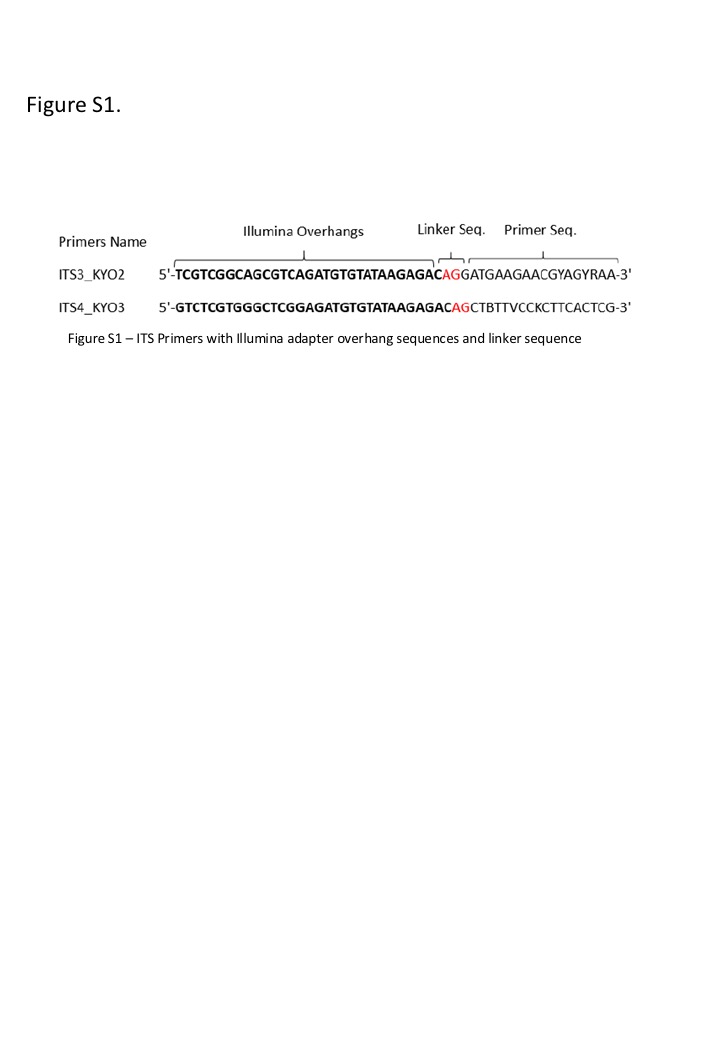


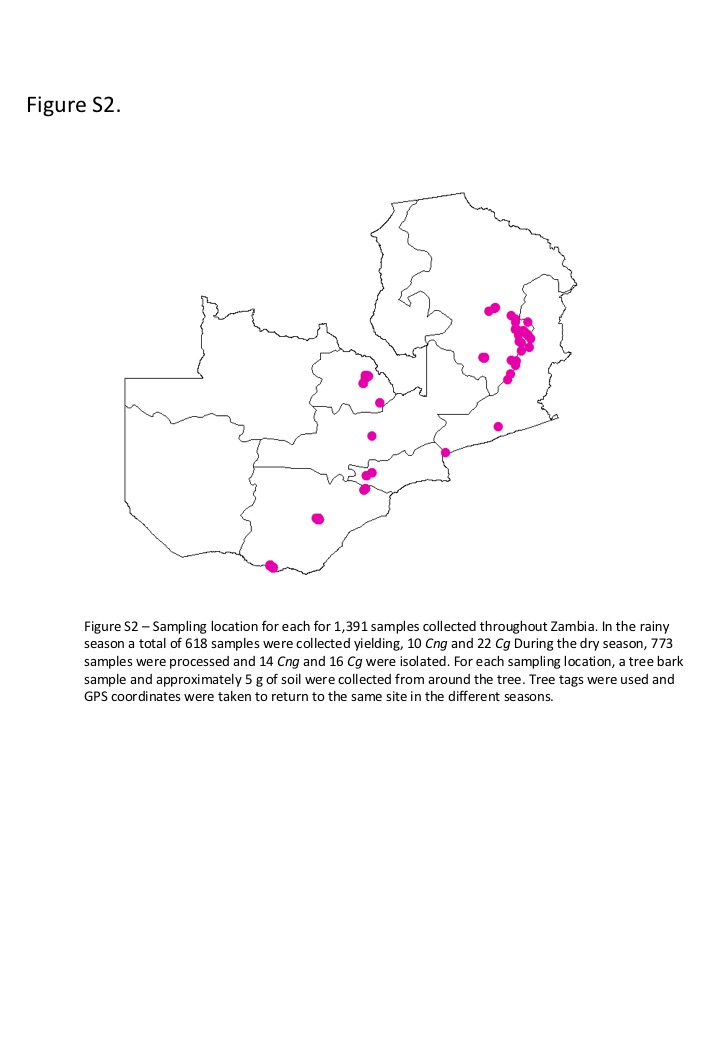


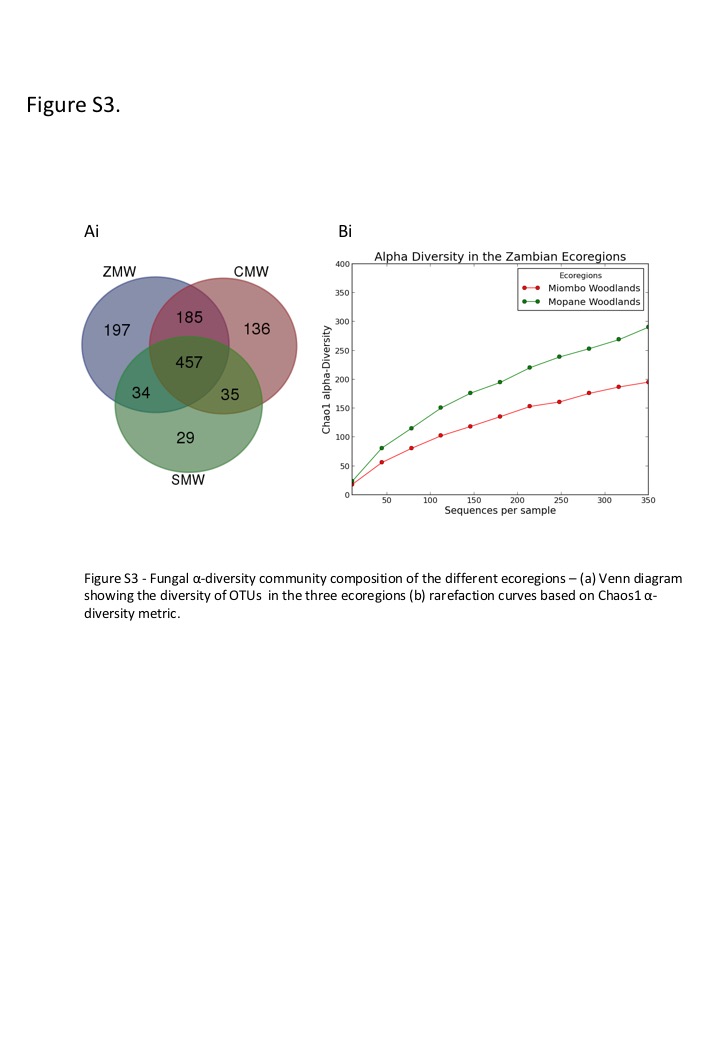


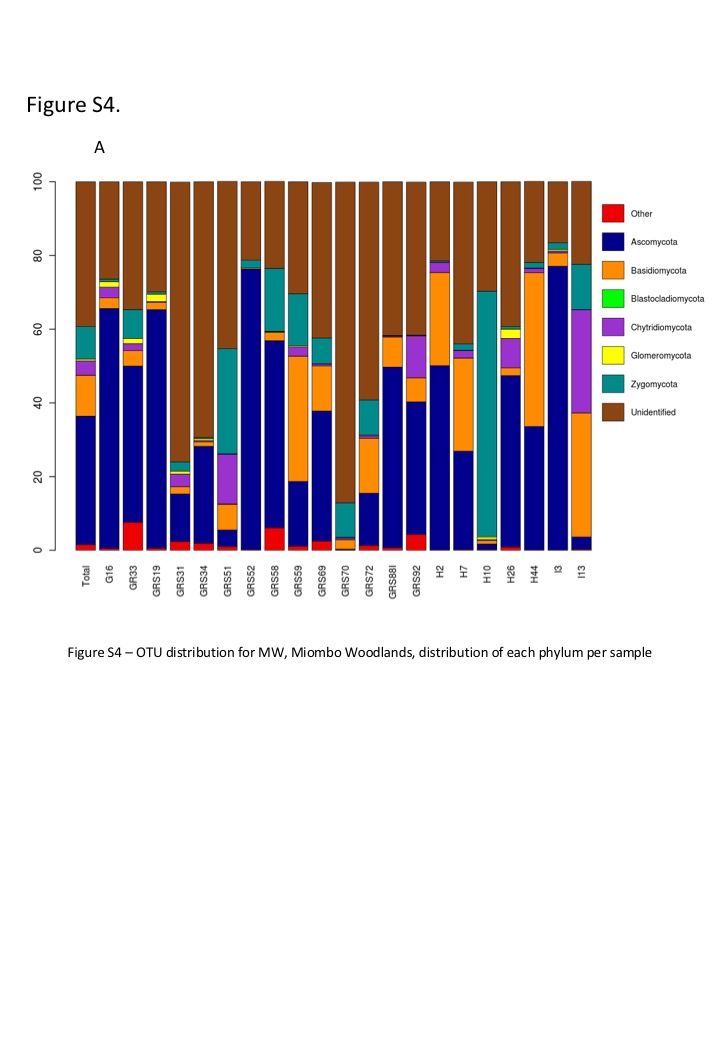


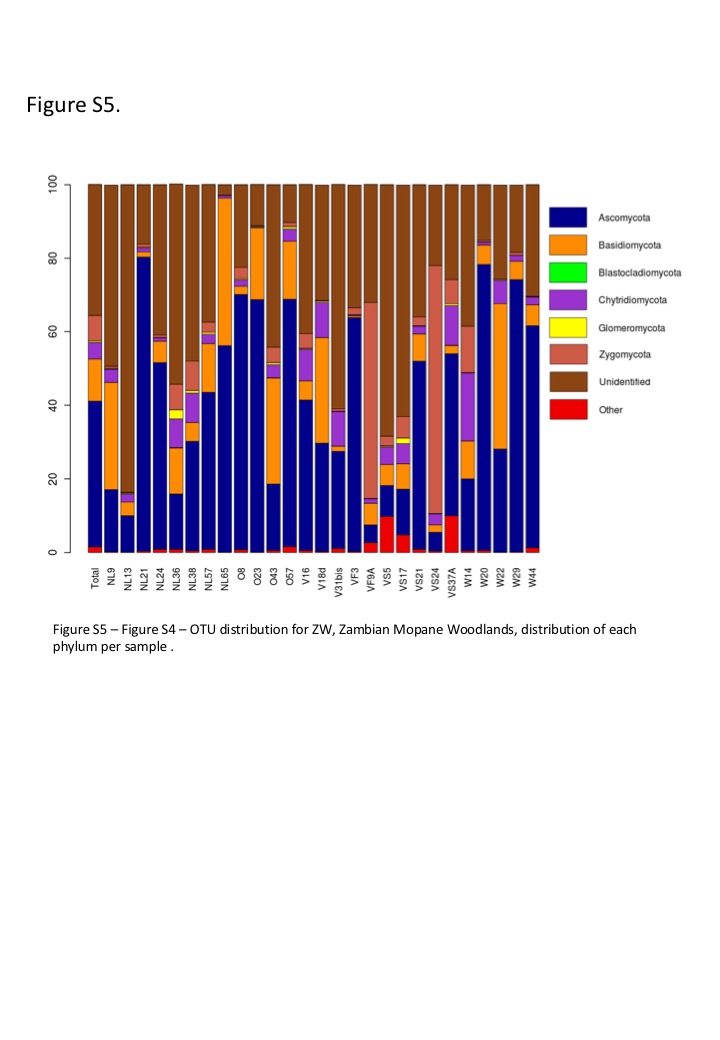


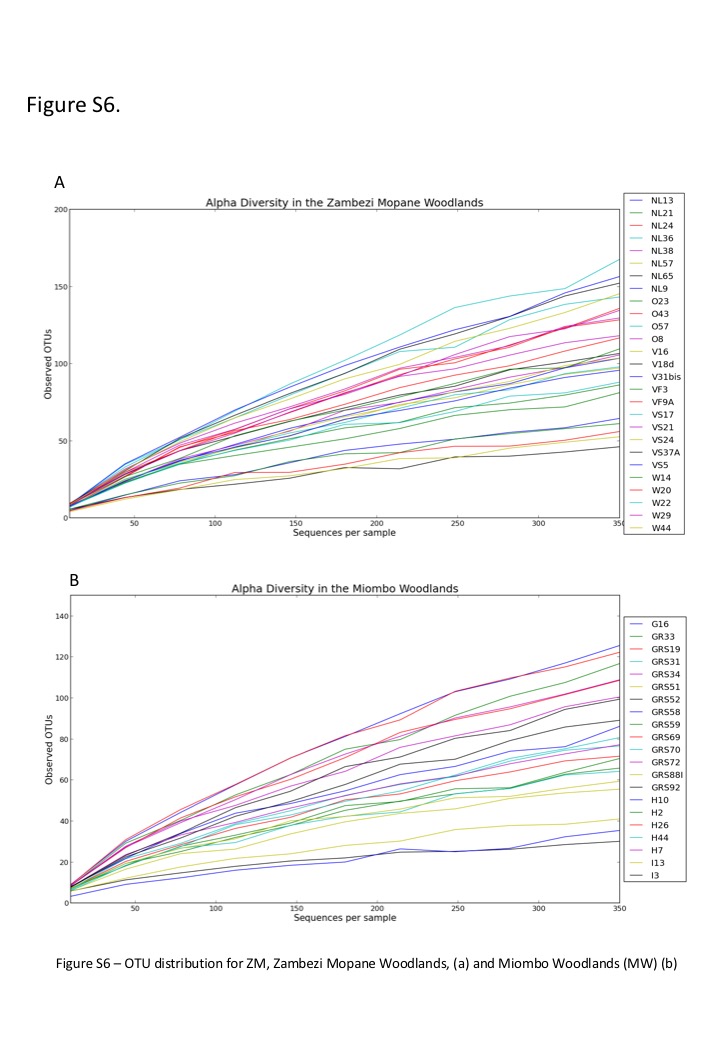


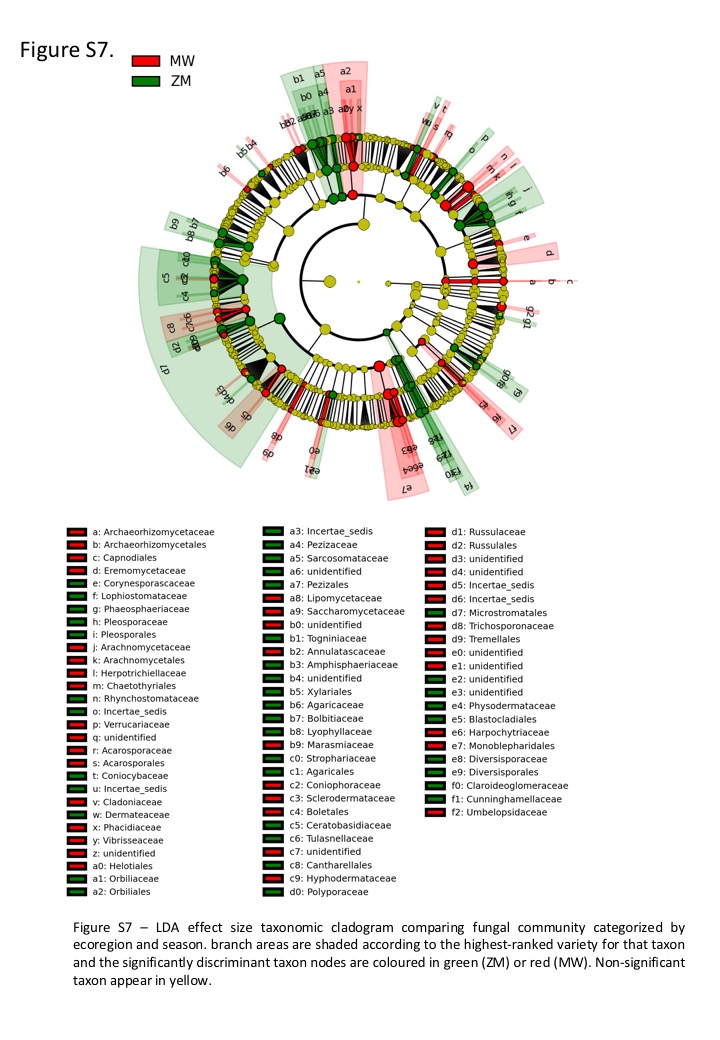


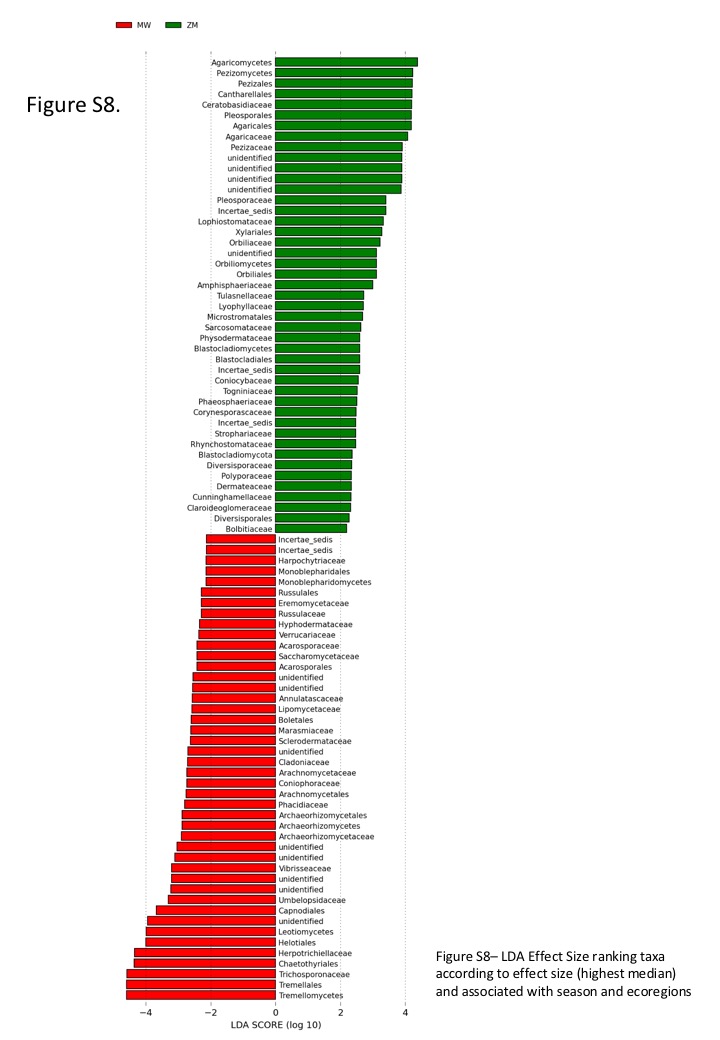


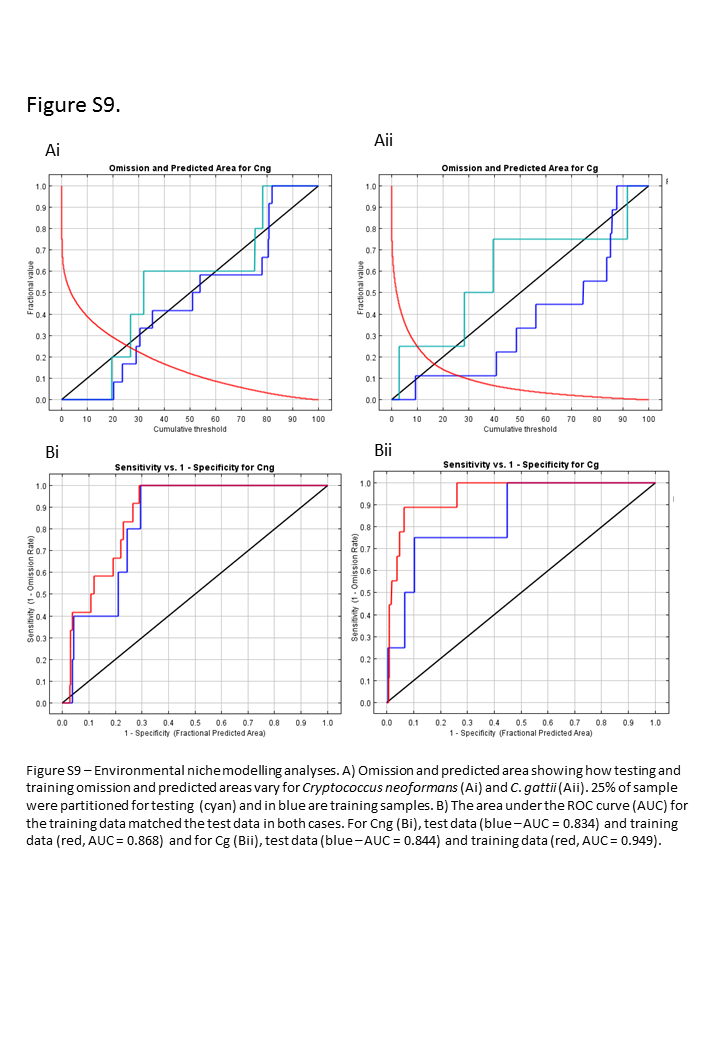

Supplement: Supplementary file 1 — Appendix S1 Results. Table S1 Relative contribution of Bioclim layers to the maxent model. Table S2 List of sample used in ITS2 metabarcoding – The two ecoregions investigated were the Zambezi Mopane Woodlands (ZM) and the Miombo Woodlands (MW). Table S3 ANOSIM and permutation manova of microbial diversity patterns across Zambian ecoregions. Table S4 Microbial patterns within Ecoregions across seasons. Table S5 Permutational manova of environmental effects on microbial diversity patterns between regions. Table S6 BEST analysis using all ecoregion. Table S7 BEST analysis in Zambezi Mopane Woodlands. Table S8 BEST analysis Central Miombo Woodlands. Table S9 Environmental and clinical isolates collected in Zambia. Table S10 Shared SNPs between lineages and group. Table S11 Uniquely shared SNPs between lineages and group – single‐nucleotide polymorphism which could only be found the two groups compared. Table S12 Cryptococcus gattii (n = 38) recovery in Zambia. Table S13 Genetic diversity among the different Cryptococcus groups. Fig. S1 ITS primers with Illumina adapter overhang sequences and linker sequence. Fig. S2 Sampling location for each for 1391 samples collected throughout Zambia. Fig. S3 Fungal α‐diversity community composition of the different ecoregions – (a) Venn diagram showing the diversity of OTUs in the three ecoregions (b) rarefaction curves based on Chaos1 α‐diversity metric. Fig. S4 OTU distribution for MW, Miombo Woodlands, distribution of each phylum per sample. Fig. S5 OTU distribution for ZW, Zambian Mopane Woodlands, distribution of each phylum per sample. Fig. S6 OTU distribution for ZW, Zambezi Mopane Woodlands, (a) and Miombo Woodlands (MW) (b). Fig. S7 LDA effect size taxonomic cladogram comparing fungal community categorized by ecoregion and season. Fig. S8 LDA effect size ranking taxa according to effect size (highest median) and associated with season and ecoregions. Fig. S9 Environmental niche modelling analyses. [file MEC-26-1991-s001.docx]
